# MUUMI: an R package for statistical and network-based meta-analysis for multi-omics data integration

**DOI:** 10.1186/s12859-026-06394-3

**Published:** 2026-02-03

**Authors:** Simo Inkala, Michele Fratello, Giusy del Giudice, Giorgia Migliaccio, Angela Serra, Dario Greco, Antonio Federico

**Affiliations:** 1 Finnish Hub for Development and Validation of Integrated Approaches (FHAIVE), Faculty of Medicine and Health Technology, Tampere University, Tampere, 33100 Finland; 2 Division of Pharmaceutical Biosciences, Faculty of Pharmacy, University of Helsinki, Helsinki, 00100 Finland

**Keywords:** Data integration, Statistical meta-analysis, Biological networks, Network analysis, Complex diseases, Multi-omics data, Systems biology

## Abstract

**Background:**

Disentangling physiopathological mechanisms of biological systems through high-level integration of omics data has become a standard procedure in life sciences. However, platform heterogeneity, batch effects, and the lack of unified methods for single- and multi-omics analyses represent relevant drawbacks that hinder the extrapolation of a meaningful biological interpretation. While statistical meta-analysis is widely used to integrate several omics datasets of the same type, it does not allow the integration of multi-modal data deriving from multi-omics experiments. Network science is at the forefront of systems biology, where the inference of molecular interactomes allowed the investigation of perturbed biological systems, by shedding light on the disrupted relationships that keep the homeostasis of complex systems.

**Results:**

Here, we present MUUMI, an R package that unifies statistical meta-analysis and network-based omics data integration within a single analytical framework. MUUMI allows the identification of robust molecular signatures through multiple meta-analytical methods, inference and analysis of molecular interactomes and the integration of multiple omics layers through similarity network fusion. We demonstrate the functionalities of MUUMI by presenting two case studies in which we analysed (1) 17 transcriptomic datasets on idiopathic pulmonary fibrosis (IPF) from both microarray and RNA-Seq platforms and (2) multi-omics data of THP-1 macrophages exposed to different polarising stimuli. In both examples, MUUMI revealed biologically coherent signatures, underscoring its value in elucidating complex biological processes.

**Conclusions:**

MUUMI leverages omics data meta-analysis, integration and interpretation that implements both traditional and network-based approaches to unleash the power of multi-study datasets. Statistical and network-based approaches are integrated in a unique framework, allowing the user to derive robust and biologically meaningful results from different studies and datasets. MUUMI is an open-source package and is freely available at https://github.com/fhaive/muumi.

**Supplementary Information:**

The online version contains supplementary material available at 10.1186/s12859-026-06394-3.

## Background

Omics data integration has long posed substantial challenges, including substantial variability in data quality, lack of standardization, and the inherent heterogeneity of platforms and data formats. These issues can introduce noise, bias, and limited interoperability, complicating efforts to combine results across multiple studies. Remarkable efforts have been carried out in order to make omics data more integrable [1]. Nevertheless, methods for interpreting integrated omics data remain scattered, and the research community still lacks a comprehensive resource that unifies both data integration and downstream interpretation.

Statistical meta-analysis has for long represented a crucial method to extrapolate evidence through the integration of independent studies addressing the same research question, aiming to generate a quantitative estimate of the phenomenon under consideration. Meta-analysis can be performed at the aggregate data (AD) level, pooling summary statistics for each study or at the individual participant data (IPD) level, integrating raw data for each participant. While IPD meta-analyses are often considered ideal, practical limitations such as data availability, intellectual property constraints, and high costs continue to make AD-based meta-analysis the most widely adopted approach [2]. Network science provides a complementary and increasingly popular framework for deciphering the intricate relationships among genes, proteins, or metabolites in complex biological systems [3]. The inference of these molecular interactomes finds diverse applications, including biomarker discovery, patient stratification, and druggability assessment. By shifting the focus from individual molecular entities to broader connectivity patterns, network-based approaches can reveal functional modules, regulatory relationships, and disease-associated sub-networks that may be overlooked by traditional methods. While network modelling has been widely utilised to integrate multi-view biological datasets [1], its potential in supporting and complementing traditional meta-analytical approaches has not yet fully exploited.

In this study, we developed MUUMI, an R package designed to streamline omics data integration and interpretation through both statistical and network-based meta-analysis. MUUMI combines multiple meta-analysis techniques within a unified pipeline, complemented by tools for network inference, community detection, and functional annotation. Moreover, it supports multi-omics data integration through Similarity Network Fusion [1], extrapolating molecular signals across distinct omics layers. We showcase the functionalities of MUUMI in two case studies: [1] a single-omics, multi-study integrative analysis of idiopathic pulmonary fibrosis (IPF) datasets, and [2] a multi-omics dataset investigating macrophage polarization. MUUMI’s R functions are made publicly available and the users can build a completely tailored pipeline, by adapting it to the specific biological problem under investigation.

## Implementation

MUUMI proposes a toolbox that integrates innovative methods in each of its components. First, we combined different meta-analytical approaches in an ensemble method for the extrapolation of robust molecular signatures from omics data of the same type. Second, we developed a novel data-driven feature selection method to prioritise biologically meaningful features obtained from the meta-analysis. Third, the toolbox incorporates an effective method for mitigating batch effects that typically arise from integrating datasets originating from different studies. Fourth, we provide an extensive suite of functions for the inference, manipulation, analysis, and interpretation of molecular interactomes. Overall, MUUMI seamlessly integrates both early- and late-stage data integration techniques within a unified framework. It addresses both horizontal and vertical integration challenges while optimizing performance by minimizing technical noise introduced through cross-study dataset integration.

The MUUMI package comprises four main modules. Module 1 focuses on statistical meta-analysis, allowing the user to pool differential analysis results from multiple independent studies addressing the same biological question. It produces a ranked list of molecular entities (e.g., genes, proteins, etc.) by integrating three distinct meta-analytical approaches: effect size, p-value-based, and rank product. The functions to run the meta-analysis with such methods are *calc_effect_size_rank*, *calc_pvalue_based_rank* and *calc_rank_base_rank*, respectively. The effect size method accounts for both within- and between-study variability [4], while Fisher’s sum of logs method aggregates p-values from multiple independent studies [5]. Rank product, on the other hand, is a non-parametric statistical approach that merges differential analysis outcomes from individual studies based on within-study ranking of molecular entities; these ranks are then combined via a one-class analysis of the rank-product statistic [6], [7]. Beyond these individual methods, MUUMI provides the opportunity to run an ensemble of these methods to increase the robustness, comprehensiveness, statistical power, and reproducibility of the analysis through the function *run_ensembl_metanalysis*. Integrating rank information from the three methods by performing the ensemble meta-analysis offers several benefits. Using multiple methods can increase the statistical power of the analysis, providing robustness to the results. Moreover, by utilizing this approach, it is possible to identify genes that are genuinely associated with the phenotype of interest, increasing the confidence of their biological relevance and the reproducibility of the results. Module 1 also includes a newly developed feature selection function that derives a threshold for the meta-analysis rank based on the biological significance of molecular entities. This is accomplished through a Gene Set Enrichment Analysis (GSEA)-based approach, which calculates enrichment scores of ranked entities over pathway-specific gene sets. The resulting threshold provides a biologically informed cutoff for selecting features for downstream analyses. The functions implemented in MUUMI to perform the GSEA analysis and the subsequent threshold computation are *compute_gsea* and *compute_gsea_thresh*, respectively.

Module 2 is dedicated to network analysis and inference. Since omics datasets commonly originate from multiple sources and experimental platforms, batch effects can hide genuine biological signals. MUUMI addresses this challenge with a function named *multi_studies_adjust*, that aggregates, scales, and mitigates batch effects, producing an integrated matrix for use in further analyses, such as network inference. Subsequently, users can infer robust biological networks using an ensemble of methods [8]. In detail, we developed the *calculate_correlation_matrix* function, which can be used to compute the correlation between genes based on their previously adjusted expression levels. The function calculates the dependence between such gene expression levels by computing parametric, non-parametric and mutual information measures, starting from a gene expression table as an input. The *calculate_correlation_matrix* function is used to combine information from different methods that analyze gene expression or other omics data to create a robust molecular network deriving from an ensemble of methods. Such a network is inferred by ranking the relationships between genes based on their importance or strength. Various analyses can then be performed on these inferred networks, including community detection via walktrap, louvain, spinglass, or the greedy algorithm through the *get_modules* function, as well as module-specific functional annotation and visualization by using the *get_reactome_from_modules* function.

Module 3 facilitates single-omics data integration derived from diverse platforms and technologies (e.g., RNA-Seq and microarray data). This module enables the aggregation of networks obtained from different single-omics views. Each omic dataset undergoes its own single-omic network analysis workflow, producing modality-specific modules. To aggregate these modules across omics, MUUMI constructs an aggregated membership matrix, where rows represent genes measured across views and columns represent modules identified in individual omics. Non-negative Matrix Factorization (NMF[9]) is then applied to this matrix through the function *aggregate_communities* to uncover latent factors that define meta-modules spanning multiple omics [10]. This decomposition enables the assessment of gene membership in integrated modules, the contribution of each original module to meta-modules, and the relative influence of entire modalities. In fact, MUUMI quantifies the relative contributions of each dataset to the aggregated communities derived from the NMF process through the function *compute_view_contributions*. Such a function groups the input communities by dataset origin, computes the contribution of each dataset to each aggregated community, and normalizes these contributions to account for total community weights. This step provides insights into how much each dataset informs the overall aggregation.

By operating on network memberships rather than raw measurements, this approach sidesteps issues related to heterogeneous data distributions and measurement platforms, while remaining computationally efficient and easily parallelisable. However, it primarily captures global similarities between single-omic modules and does not account for connectivity patterns across networks.

Finally, Module 4 extends the integration paradigm to a multi-omics setting through the Similarity Network Fusion (SNF) algorithm [1]. We have built a SNF-wrapper function named *snf_based_integration* that constructs a similarity network for each omics layer (e.g., transcriptomics, proteomics, epigenomics) and iteratively fuses these into a single consensus network. This unified representation highlights relationships consistent across multiple data types. The rationale for embedding SNF in our framework lies in the integrability of its results with other functionalities of MUUMI. SNF infers a similarity network for each layer of omics data, and fusing them in order to obtain a fused similarity network that recapitulates the molecular make-up of the biological system under study. The fused network is ready for downstream analyses, such as community detection followed by functional annotation, topological analyses, Edge Set Enrichment Analysis (ESEA).

**Fig. 1:**
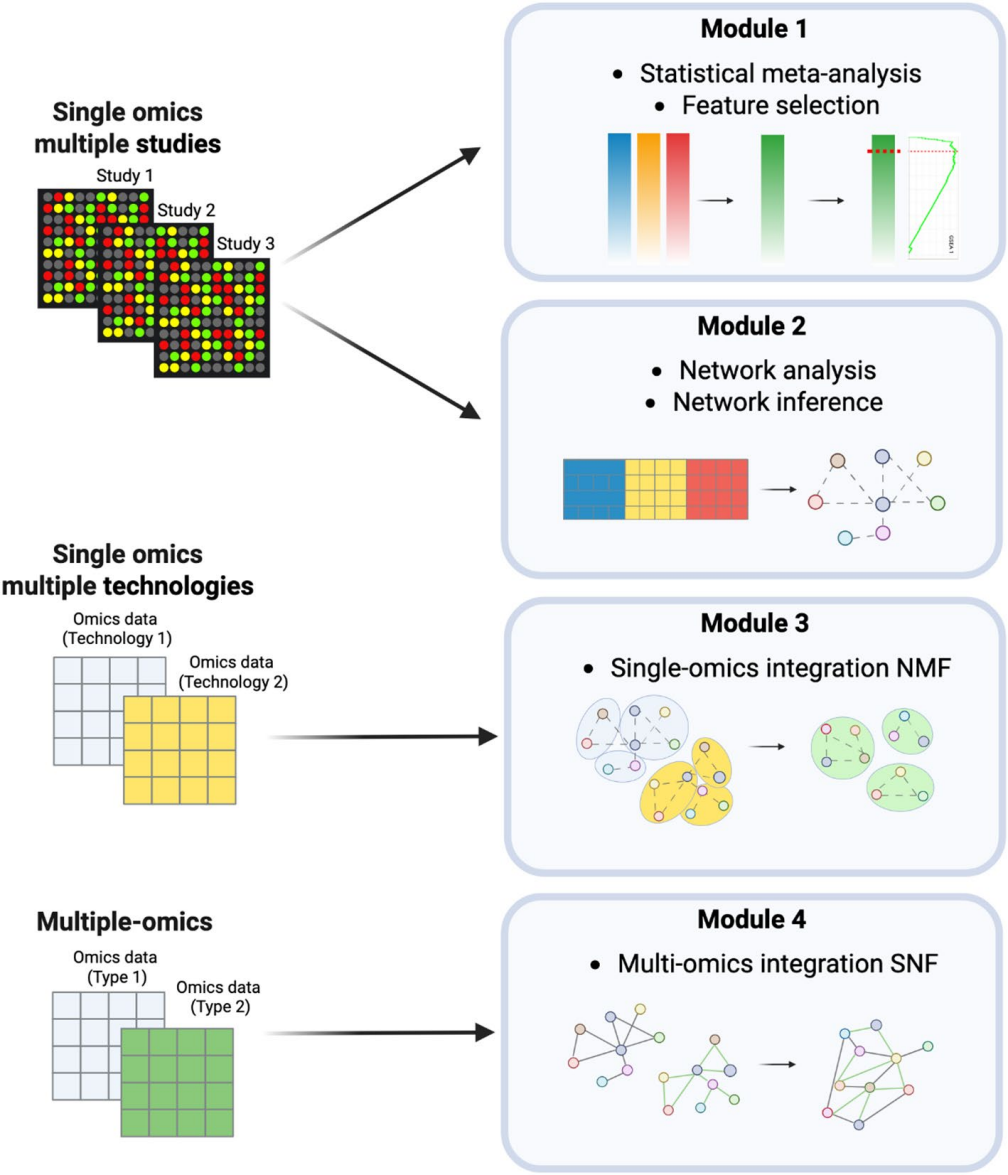
Overview of the functionalities of the MUUMI package

Therefore, to further assist in the interpretation and visualization of constructed networks, this final module also supports overrepresentation analysis (ORA) on network communities and a topological enrichment approach based on Edge Set Enrichment Analysis (ESEA) through the function *compute_esea*. Results can be visualized at both the individual and comparative levels for single or multiple networks, facilitating a deeper understanding of complex molecular interactions and enriched functional pathways. Figure 1 shows the functionalities of the MUUMI package. Detailed information about the implementation and functions included in MUUMI can be found in Additional file 1.

## Results

### Case Study 1: Meta-analysis and network integration of transcriptomics data reveal molecular determinants of idiopathic pulmonary fibrosis

#### Experimental setup and data used

To showcase the functionalities of MUUMI, we performed a case study on idiopathic pulmonary fibrosis (IPF) by analysing samples deriving from the integration of 17 pre-processed and harmonized public transcriptomics datasets. Among all the included datasets, 11 derive from RNA sequencing (RNA-Seq) while 6 from microarray experiments (Table 1). The collected data include both fibrotic lung biopsy samples of IPF patients and biopsy samples of healthy individuals. Datasets are made publicly available in Zenodo (*https://zenodo.org/doi/*10.5281/zenodo.10692128*).* We employed ESPERANTO, a R/Shiny application, to efficiently curate and harmonize metadata using its semi-supervised approach, streamlining the metadata curation process [11]. This case study aims to perform the integration of single omics datasets and carry out both statistical and network-based meta-analysis. The resulting data can then be utilised to uncover molecular vulnerabilities and dysregulated biological processes underlying the IPF phenotype.

**Table 1:** Publicly available transcriptomics datasets included in the case study

Repository	GEO dataset ID	Platform	Citation	Number of IPF samples	Number of healthy samples
NCBI Gene Expression Omnibus (GEO)	GSE150910	Illumina NovaSeq 6000	[12]	103	103
NCBI Gene Expression Omnibus (GEO)	GSE213001	Illumina HiSeq 3000	[13]	62	41
NCBI Gene Expression Omnibus (GEO)	GSE124685	Ion Torrent Proton	[14]	49	35
NCBI Gene Expression Omnibus (GEO)	GSE199949	Illumina HiSeq 4000	[15]	26	16
NCBI Gene Expression Omnibus (GEO)	GSE92592	Illumina HiSeq 2000	[16]	20	19
NCBI Gene Expression Omnibus (GEO)	GSE199152	Illumina HiSeq 2500	Unpublished	20	4
NCBI Gene Expression Omnibus (GEO)	GSE53845	Agilent-014850 4 × 44 K G4112F	[17]	40	8
NCBI Gene Expression Omnibus (GEO)	GSE166036	Illumina HiSeq 4000	[18]	10	4
NCBI Gene Expression Omnibus (GEO)	GSE184316	Ion Torrent Proton	Unpublished	40	24
NCBI Gene Expression Omnibus (GEO)	GSE110147	Affymetrix 1.0 ST Array	[19]	22	11
NCBI Gene Expression Omnibus (GEO)	GSE21369	Affymetrix U133 Plus 2.0	[20]	11	6
NCBI Gene Expression Omnibus (GEO)	GSE24206	Affymetrix U133 Plus 2.0 Array	[21]	17	6
NCBI Gene Expression Omnibus (GEO)	GSE72073	Affymetrix Transcriptome Array 2.0	[22]	5	3
NCBI Gene Expression Omnibus (GEO)	GSE169500	Ion Torrent PGM	[23]	20	10
NCBI Gene Expression Omnibus (GEO)	GSE99621	Illumina HiSeq 2500	[24]	18	8
NCBI Gene Expression Omnibus (GEO)	GSE10667	Agilent-014850 4 × 44 K G4112F	[25]	31	15
NCBI Gene Expression Omnibus (GEO)	GSE138283	Illumina NextSeq 550	[26]	12	5

### Phenotypic and molecular basis of IPF

IPF is a chronic, progressive, and age-related interstitial lung disease (ILD) that is irreversible and typically fatal. IPF has an unknown origin and only a few treatment options are currently available that are limited in their efficacy [27]. In IPF, progressive formation of scar tissue in the supportive interstitium of the lungs occurs, causing breathing to become increasingly difficult [28]. Histopathologically IPF typically exhibits subpleural and paraseptal fibrosis, honeycombing, and regions of less affected and normal parenchyma [27]. The proposed pathogenesis connects IPF to a lung susceptible to aging-related changes, which is subject to repetitive alveolar injuries triggered by factors such as inhaled cigarette smoke, microaspiration, nanomaterials, gastroesophageal reflux, or viruses [27], [29]. These injuries can lead to type I and type II epithelial cell death. Following micro-injuries and epithelial cell apoptosis, there is an increased vascular permeability to proteins like fibrinogen and fibronectin, leading to the formation of a wound clot. Subsequently, there is migration and proliferation of bronchiolar and alveolar epithelial cells, representing a rapid response of the respiratory tissue attempting self-repair. Abnormally activated epithelial cells release various chemokines, cytokines, and epidermal growth factors, attracting fibroblasts, immune system cells such as alveolar macrophages, and monocytes that differentiate into macrophages. Moreover, these cells secrete TGF-β1, promoting epithelial-mesenchymal transition, extracellular matrix remodeling, and fibroblast differentiation into myofibroblasts. The heterogeneous macrophage population also secretes chemokines and growth factors like TGF-β, inducing the growth of fibrotic tissue in the extracellular matrix. Positive feedback loops contribute to the progressive expansion of the fibrotic tissue [30–32].

### Identification of robust IPF genes by statistical meta-analysis

For each dataset indicated in Table 1, we conducted differential gene expression analysis between IPF and healthy samples. For RNA-Seq datasets, gene expression data normalisation and differential gene expression analysis were performed using DESeq2 version 1.24.0 [33]. DNA microarray data preprocessing was carried out by using the eUTOPIA software, which implements the limma package for expression value normalisation and performed differential gene expression analysis by applying quantile normalisation and linear model [34, 35]. We adjusted nominal p-values in both RNA-Seq and microarray datasets by using the Benjamini & Hochberg method [36]. In order to identify robust IPF-associated genes, we integrated the results of the differential expression analysis by performing a statistical-based meta-analysis utilising the function *run_ensembl_metanalysis*, whose input consists of adjusted *p*-values from all the datasets, with gene symbols as row names. All three methods included in our pipeline, namely “effect_size,” “pvalue,” and “rank_product,” were utilized in the ensemble analysis. 10,460 genes were detected as expressed in RNASeq, while 14,437 in DNA microarray experiments. By selecting common genes measured by both of the platforms, 9,371 genes were included in the analysis (representing 89,6% and 65% of all the detected genes, respectively). The full meta-analysis gene rank is available at https://zenodo.org/records/15019060.

Among the highest-ranked genes, we identified several members of the *COL* family: *COL3A1*, which holds the highest overall ranking in the analysis, along with *COL14A1*,* COL15A1*, and *COL1A2* which are also in the top 20. The transcriptional profile of lung fibroblasts can influence the extracellular matrix (ECM) proteins encoded by these genes, leading to alterations in the ECM of lungs affected by IPF [37]. Accumulating evidence suggests that the mechanical interactions between fibroblasts and the stiffened ECM create a feedforward mechanism, contributing to the persistence and progression of pulmonary fibrosis [38, 39]. A study by Huang, S. et al. 2022 [40] revealed that Asporin (ASPN) promotes the differentiation of lung myofibroblasts induced by TGF-β by facilitating Rab11-dependent recycling of TβRI. ASPN, a member of the small leucine-rich proteoglycan family known for its crucial roles in tissue injury and regeneration, is highly expressed in various tumor types and has the capacity to upregulate TGF-β1. TGF-β induces fibrotic tissue growth in the ECM [41]. Elevated protein levels of MMP7 have been observed in IPF compared to samples from healthy controls, and it serves as a predictive biomarker for disease progression in IPF [42]. In individuals affected by IPF, the expression of matrix metalloproteinases (MMPs) is dysregulated, resulting in substantial architectural remodeling in the lung microenvironment. MMPs have been considered potential therapeutic targets for IPF [43]. Yu, G., et al. 2018 [44] found that the activity and expression of iodothyronine deiodinase 2 (DIO2), was higher in the lungs of patients with IPF compared to control individuals and correlated with disease severity. Based on these findings, we can confirm that the genes ranked by the *run_ensembl_metanalysis* function hold biological relevance in IPF, indicating the efficacy of our approach in prioritizing genes resulting from differential analysis of multiple omics datasets.

### Identification of functionally relevant genes and pathways derived from statistical-based meta-analysis

We performed Gene Set Enrichment Analysis (GSEA) utilizing the function *compute_gsea*. The background gene universe utilised for this analysis is the entire ranked gene list that was obtained from the meta-analysis, composed of 9,371 genes. Reactome pathways from the Molecular Signatures Database (MSigDB) [45] C2 collection were used. To ensure meaningful results, the minimum pathway size was set to 10 genes and the number of permutations to 100,000. The 15 most enriched pathways are represented in Table 2.

**Table 2:** Top 15 enriched reactome pathways in GSEA analysis

Pathway	*p*-val	*p*-adj	ES	NES	nMoreExtreme	Size
Extracellular matrix organization	1.00E-05	0.003423	0.458414	1.683331	0	185
Regulation of insulin like growth factor IGF transport and uptake by insulin like growth factor binding proteins IGFBPs	1.00E-05	0.003423	0.502973	1.716711	0	64
Collagen degradation	1.00E-05	0.003423	0.587627	1.894219	0	37
Assembly of collagen fibrils and other multimeric structures	2.00E-05	0.005132	0.537653	1.763733	1	43
ECM proteoglycans	4.00E-05	0.007379	0.512437	1.704437	3	49
Collagen biosynthesis and modifying enzymes	5.00E-05	0.007379	0.527783	1.731356	4	43
Molecules associated with elastic fibres	5.03E-05	0.007379	0.633992	1.930158	4	24
Degradation of the extracellular matrix	7.00E-05	0.00802	0.454145	1.58119	6	81
Collagen chain trimerization	7.03E-05	0.00802	0.603954	1.860309	6	26
Reactome collagen Formation	8.00E-05	0.008208	0.482114	1.63859	7	61
Metabolism of steroids	9.00E-05	0.008394	0.449728	1.573069	8	86
Integrin cell surface interactions	1.10E-04	0.009405	0.489075	1.642485	10	54
Elastic fibre formation	1.60E-04	0.012312	0.565026	1.774937	15	30
Class A1 rhodopsin like receptors	1.70E-04	0.012312	0.438244	1.536992	16	89
GPRC ligand binding	1.80E-04	0.012312	0.405372	1.454706	17	124

The *compute_gsea_thresh* function computes a threshold for the statistical meta-analysis gene rank based on the GSEA enrichment score. In this case study, the threshold was set to the top 2860 genes out of 9371 genes.

The GSEA analysis results of MUUMI (Table 2) successfully identified significant functional pathways associated with IPF. Notably, pathways related to ECM remodeling were found to be significantly enriched among the ranked genes in the meta-analysis. This emphasizes the prevalent occurrence of ECM remodeling in lung diseases such as IPF [46, 47]. “Extracellular Matrix Organization” and “Collagen Formation” pathways highlight the structural aspects of the ECM, emphasizing the importance of collagen assembly and fibrillogenesis in the pathogenesis of IPF. Additionally, pathways such as “Collagen Degradation” and “Degradation of The Extracellular Matrix” imply dysregulation in the turnover of ECM components, possibly contributing to fibrotic changes observed in IPF. Pathways like “Regulation of Insulin-Like Growth Factor IGF Transport” and “Integrin Cell Surface Interactions” suggest potential connections between growth factor signaling and cellular adhesion, emphasizing the complex interplay between signaling pathways and fibrotic processes. Moreover, pathways related to elastic fibers and G protein-coupled receptor (GPCR) ligand binding shed light on the involvement of these structural and signaling elements in the context of IPF pathology. The enrichment of pathways associated with metabolism, such as “Metabolism of Steroids,” suggests a broader impact on cellular homeostasis. The results of the GSEA analysis demonstrate that MUUMI is capable of recapitulating biologically relevant pathways based on the statistical meta-analysis.

### Mitigation of technical effects prior multi-study data integration

**Fig. 2:**
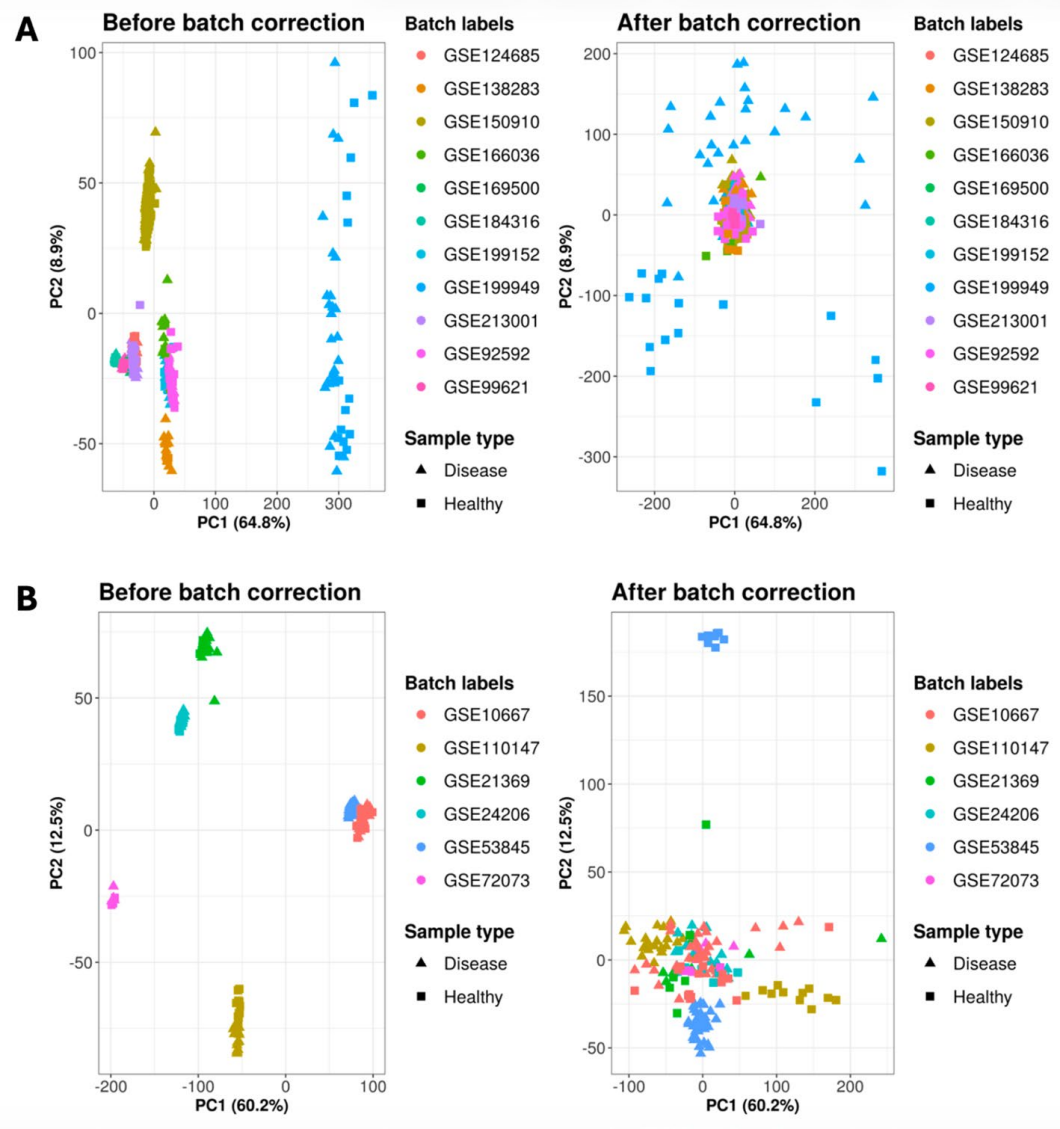
Panel A: PCA plots represent RNA-Seq samples both before and after batch correction. Panel B: PCA plots represent microarray samples before and after batch correction. In both panels, the data points are coloured according to the dataset of origin (batch labels), while the shape indicates the sample type

Technical nuisance can heavily affect the results of omics data, leading to artifacts in the results and their interpretation. Such a phenomenon is known as batch effect. Batch effect can derive from protocols, reagents, equipment, laboratory conditions, sample collection, storage, preparation methods, time, and so forth. Therefore, since gene expression datasets utilised in our case study derive from different studies, it is essential to mitigate the batch effect before carrying out subsequent integrative analyses, such as inferring network relationships [48]. To carry out batch adjustment, considering the diverse origins of the gene expression datasets, it was essential to carry out the dataset integration separately for DNA microarray and RNA-Seq datasets. The batch adjustment was carried out with the *multi_studies_adjust* function which takes as an input the integrated expression matrix, the sample labels, and the batch labels. The embedded functionality to carry out batch adjustment in the *multi_studies_adjust* utilizes the *pamr.batchadjust* from the *pamr* package [49]. Figure 2 shows the outcome of the batch effect adjustment on both RNA-Sequencing (A) and DNA microarray (B) datasets.

### Integrated datasets for network-based meta-analysis

**Fig. 3:**
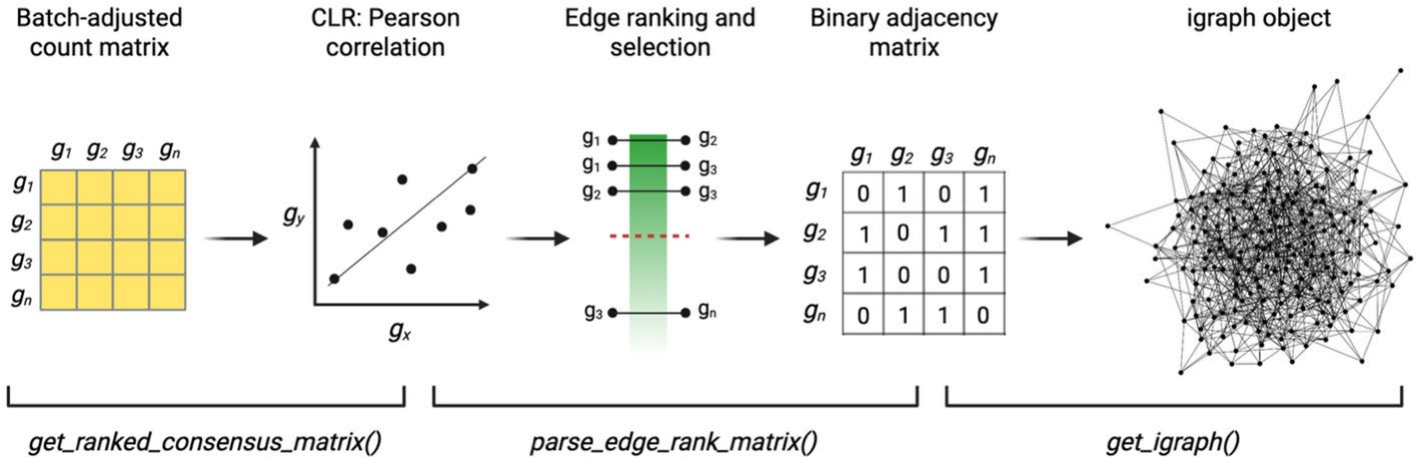
Analytical steps performed to infer gene coexpression networks through the use of MUUMI’s functions

Subsequently, we aimed to investigate co-expression patterns of the genes obtained from the meta-analysis. To do so, we inferred gene co-expression network models from IPF samples and healthy counterparts profiled through RNA-Seq datasets. The networks were constructed separately for IPF and healthy conditions to enable differential network analysis and comparative investigation. This approach is essential for identifying disease-specific interactions and biological processes. To infer the networks from the normalised and batch-adjusted read count matrices, we performed the following analytical steps:

1. We utilised the *get_ranked_consensus_matrix* function to compute pairwise correlation measures between genes, based on their expression levels for both disease and healthy samples independently. In this case study, we computed Pearson correlation to build a squared adjacency matrix where values represent network’s edge weights.
2. Therefore, we set CLR as a network inference algorithm. For each gene, CLR then models the empirical distribution of its Pearson correlation values with all other genes and converts each pairwise correlation score into a z-score relative to the gene-specific background distribution. These z-scores capture how unusual a particular association is for each gene, given its overall variability and propensity for broad correlations. CLR integrates the z-scores from both genes in a pair into a single confidence score. Edges with high combined scores are interpreted as biologically meaningful interactions. This procedure aims to suppress non-specific correlations.
3. Subsequently, we completed the network inference through the *parse_edge_rank_matrix* function, which (1) ranks edges based on their confidence score and (2) systematically adds edges to the graph from the top of the rank until all the nodes of the network are connected. The output of this function is a binary squared matrix, where 1 indicates the presence of an edge between two nodes while 0 indicates that no edge is present between two nodes.
4. We converted the binary adjacency matrix to an igraph object using the *get_iGraph* function. The number of vertices and edges in each network are reported in Table 3. The general pipeline to infer coexpression networks through the use of MUUMI’s functions is outlined in Fig. 3.

### Functional characterisation of the networks

**Fig. 4:**
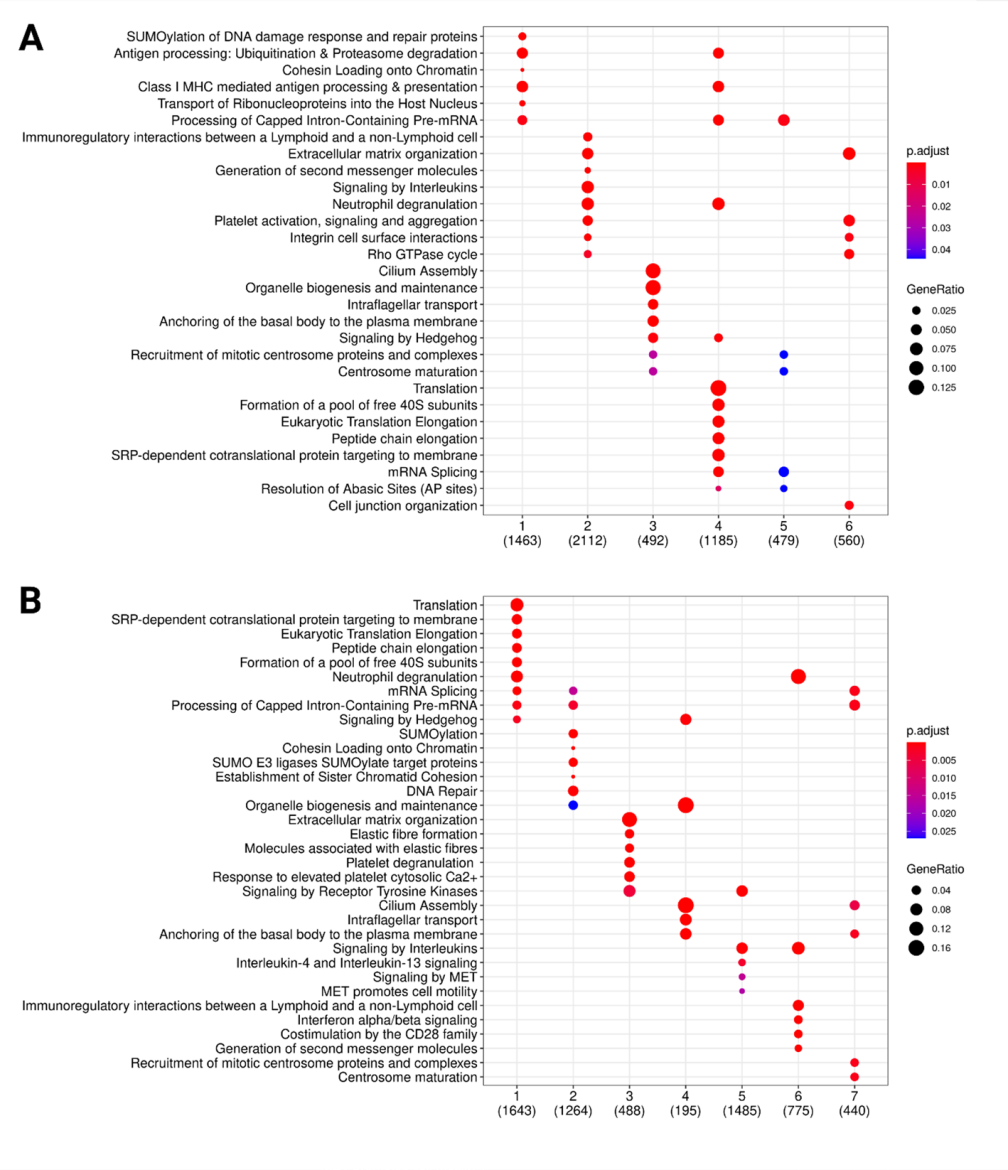
Bubble plots of significant Reactome pathways in the RNA-Seq disease network **A** and RNA-Seq healthy network **B**. X-axis represents the communities in the networks and Y-axis represents the Reactome pathways. The color of the bubble represents the significance of the pathway in the module and the size of the bubble represents the relative amount of genes in the community included in the Reactome pathway

Community detection for each network was performed using the *get_modules* function, which implements the walktrap [50], spinglass [51, 52], louvain [53] and the greedy algorithm [54]. Walktrap detects communities by assessing how short random walks propagate through the network structure. Nodes that are repeatedly visited together by these walks are considered structurally similar and are merged through hierarchical agglomeration, producing a dendrogram that reveals a community structure. This makes walktrap well suited for detecting fine-grained or nested modules and for capturing local interaction patterns. In contrast, Louvain aims to find a single partition that maximizes modularity, a global criterion that favors coarser communities and may overlook smaller but biologically meaningful clusters. Louvain is computationally efficient for very large networks, but its stochastic optimization can yield different results across runs and it does not produce a hierarchical decomposition. Spinglass methods model community detection by resembling an energy-minimization problem from statistical physics, allowing flexible partitioning but requiring intensive computation and careful parameter tuning, which makes it more suitable for small-to-medium sized networks. The greedy algorithm, by contrast, begins with each node in its own community and repeatedly merges the pair of communities that produces the greatest immediate gain in a global quality score. This greedy strategy is computationally efficient and performs well on large, sparse networks, but its reliance on locally optimal merges can cause it to overlook smaller or hierarchically nested communities. Given the size of the networks under investigation in this study and the need to characterize well defined and biologically relevant local structures, we here employed the walktrap algorithm. The number of communities in each network is shown in Table 3. In biological networks, communities represent functionally distinct, cohesive clusters of genes, working in a coordinated fashion in order to carry out a specific cellular function. Therefore, we conducted a functional annotation of each of the detected communities deriving from disease and healthy samples by using the *get_reactome_from_modules* function to identify significantly enriched biological pathways. To aggregate and visualize the pathway enrichment results of each community, we used the function *get_bubble_plot_from_pathways* to get a general visualisation of the overall functional properties of each of the analysed networks. The results of this analysis are displayed in Fig. 4.

**Table 3:** Number of modules, vertices, and edges in RNA-Seq and microarray networks

Network	Communities	Vertices	Edges
RNA-Seq disease samples	6	10,460	899,649
RNA-Seq healthy samples	8	10,460	1,373,581
Microarray disease samples	17	14,437	612,359
Microarray healthy samples	6	14,437	572,961

The network derived from the meta-analysis of RNA-sequencing data includes several gene communities associated with disease phenotype. One of the primary disease-associated communities involves antigen presentation and processing, emphasizing the role of immune system activation in fibrosis [55]. Also, immunoregulatory interactions between a Lymphoid and a non-Lymphoid cell Reactome pathway were significantly enriched in community two of the disease network. Different chemokines are connected to the occurrence of IPF, and an uneven distribution of angiogenic chemokines has been correlated with vascular remodeling in the condition [55]. Another significant module includes platelet activation, neutrophil degranulation, and extracellular compartment alterations, suggesting a strong inflammatory response and extracellular matrix remodeling, both critical in disease development. The ECM in the lungs consists of collagens, elastin, glycoproteins, and proteoglycans. These components play a central role in providing structural support for cells and ensuring the mechanical stability and elastic recoil essential for normal lung function. In IPF, a characteristic feature is the accumulation of myofibroblasts in clusters known as fibroblastic foci. This leads to a significant deposition of ECM within the interstitium, destroying lung architecture [56]. Additionally, cilia impairment has been identified as a key factor, reinforcing its relevance in various respiratory conditions. The analysis also highlights significant alterations in translation, transcription, and mRNA metabolism, indicating broad regulatory changes at the molecular level that could contribute to disease-associated gene expression alterations. Furthermore, community five underscores disruptions in cell junction integrity and its links to the extracellular functions reported in community two.

In contrast, the communities identified in healthy conditions highlight key biological processes crucial for homeostasis, including translation, transcription, and mRNA metabolism. Elastic fibers also emerge as an essential module, emphasizing their role in tissue elasticity and structural integrity. Notably, the control transcriptional profiles derive from healthy lungs, which therefore retain the majority of tissue functionalities. Cilia function remains a key feature in healthy conditions, mirroring its significance in disease when this function is altered.

Subsequently, we showcase the late integration functionalities of MUUMI by integrating transcriptomics data deriving from both RNA-Seq and DNA microarrays. First, we identified the communities in each of the inferred networks for both of the technologies. We then used the *aggregate_communities* function to independently aggregate communities from RNA-Seq and DNA microarray experiments for both disease and healthy networks. The number of aggregated communities was determined by averaging and rounding to the nearest integer based on the community counts in the RNA-Seq and DNA microarray networks (Table 3).

By incorporating DNA microarray data alongside RNA-Seq, the merged meta-analysis yielded cleaner and more consolidated results. Notably, functional redundancies observed in RNA-Seq disease communities, such as communities two and five, appear condensed into a single community, streamlining the interpretation of immune-related and extracellular processes. Previously unobserved functions, such as the impact on mitochondria, have now emerged as key elements. This finding aligns with recent research demonstrating that mitochondrial quality significantly influences pulmonary fibrosis (PF) progression [57]. Further considerations suggest that mitochondrial function plays a crucial role in disease progression. The emergence of mitochondrial dysfunction as a significant factor provides a novel perspective on pulmonary fibrosis pathogenesis. This insight is supported by recent findings highlighting mitochondrial quality as a key determinant of disease severity. Additionally, interleukin signaling, particularly IL-4 and IL-13, plays a crucial role in immune regulation and tissue maintenance. Furthermore, interferons, CD28, and other interleukins emerge as critical immune modulators maintaining a healthy physiological state.

Additionally, the role of nonsense-mediated mRNA decay (NMD) has been identified in community four, implicating various mechanisms of gene expression regulation. NMD serves as a critical surveillance pathway for controlling mRNA quality and abundance. Gene expression deregulation through various epigenetic mechanisms is a known contributor to idiopathic pulmonary fibrosis (IPF). Interestingly, NMD based approaches have recently emerged as a therapeutic target in cystic fibrosis.

**Fig. 5:**
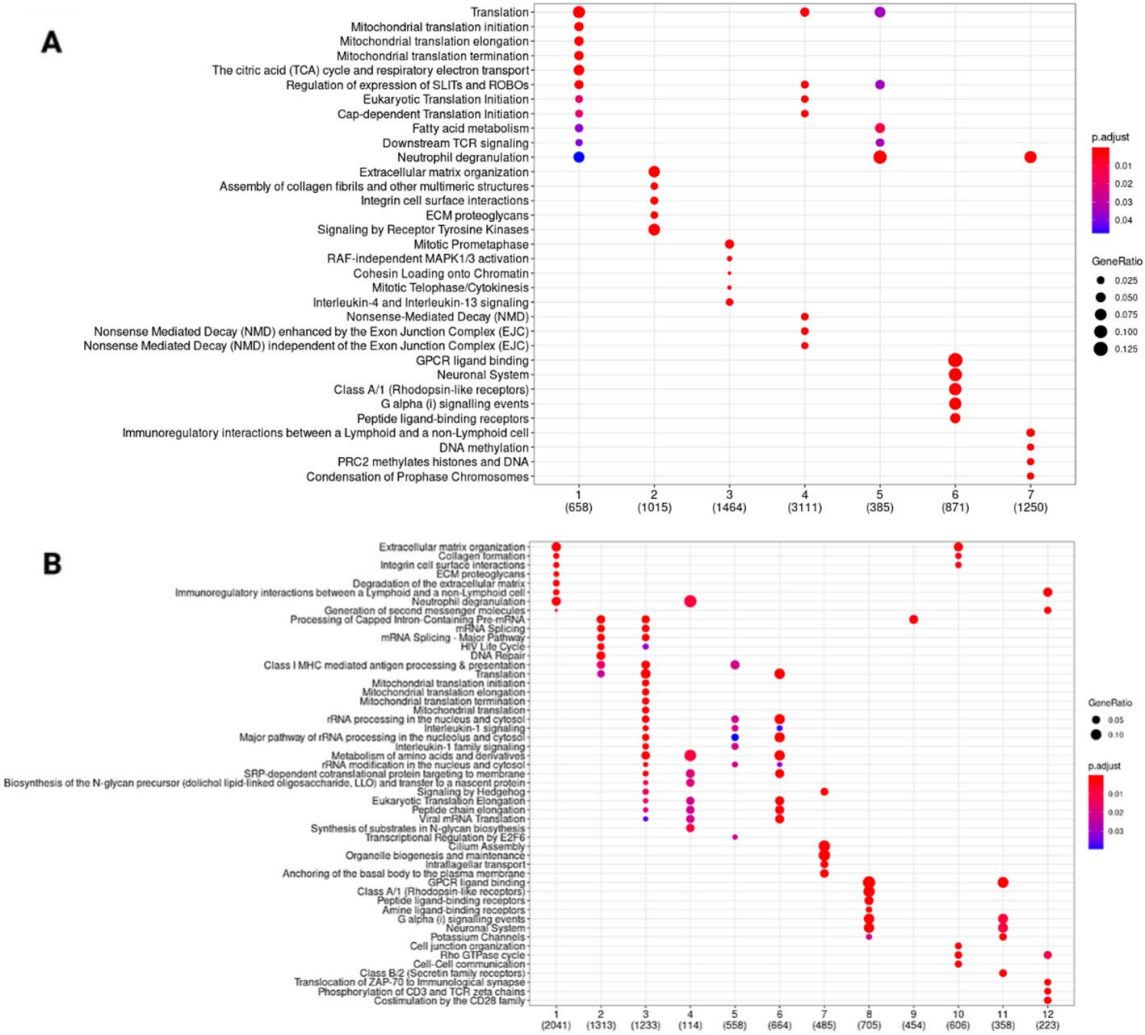
Bubble plots of significant Reactome pathways in the integrated co-expression networks deriving from both RNA-Seq and microarrays. Panel A refers to the communities deriving from IPF samples and panel B refers to communities deriving from healthy samples. X-axis represents the community numbers and Y-axis represents the enriched pathways. The color of the bubble represents the significance of the pathway in the community and the size of the bubble represents the relative amount of genes in the community that are included in the pathway

This functional interpretation offers valuable insights into the biological processes underlying disease states. The integration of multiple datasets not only refines the understanding of previously identified functionalities but also reveals novel functional associations, particularly with mitochondrial dysfunction and RNA surveillance mechanisms. Overall, the diverse array of pathways highlights the intricate molecular landscape involved in IPF, providing potential targets for therapeutic intervention and a deeper understanding of the disease mechanisms (Fig. 5).

**Fig. 6:**
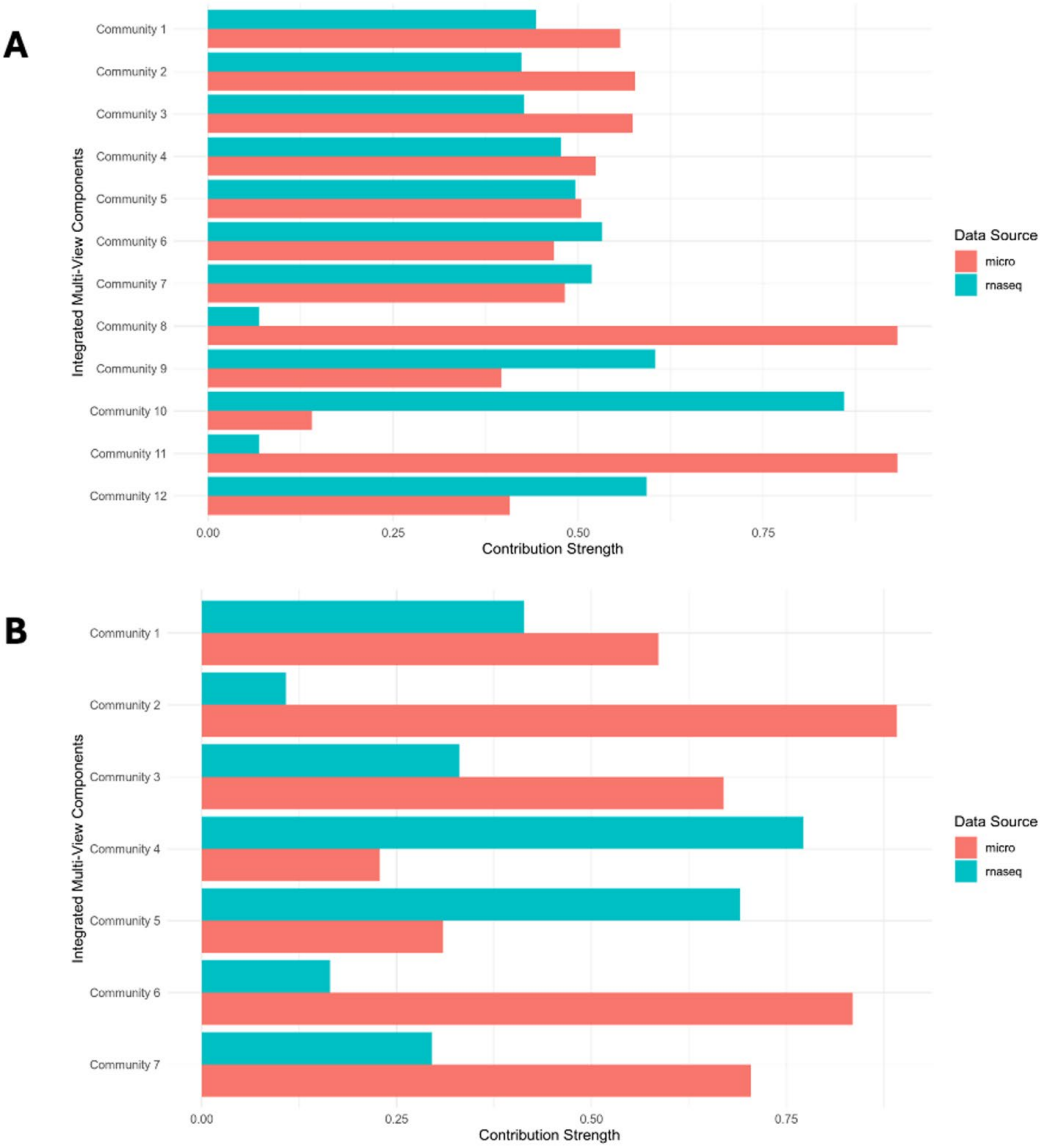
Contribution of each individual data source to the integrated communities in both disease (panel A) and healthy (panel B) samples

Finally, we computed the contributions of each of RNA-Seq and microarray technologies to the inference of the integrated communities. The contribution of each data source is calculated as the sum of its weights across all genes, normalized by the total weight for each community. These weights are derived using non-negative matrix factorization (NMF), which integrates gene communities identified in each data source and condition. Notably, some integrated communities exhibit relatively balanced contributions from all data sources (e.g., communities 1–7 in the diseased samples and community 1 in the healthy samples), indicating that these communities are shared across multiple data views. In contrast, the remaining communities show more uneven contributions, suggesting that these integrated communities are uniquely driven by a single data source (Fig. 6). These findings underscore the strength of this integrative approach in both consolidating redundant communities across different data modalities and capturing unique, dataset-specific information that would otherwise remain undetected.

### Limitations of the study

This study utilised multi-omics profiles obtained from public repositories. The availability of clinical data was usually insufficient. The limited detailed clinical information in many datasets manifests challenges to the predictive capacity of this study. It inhibits our ability to infer certain clinical parameters for patients with IPF, such as disease severity, time elapsed since the initial diagnosis, and the anatomical location of the samples. Additionally, the lack of information concerning the current administration of active pharmacological therapy to the patients is a noteworthy limitation. The transcriptional signatures shaping the topology of network models may be impacted by ongoing or terminated pharmacological therapies, potentially interfering with the pharmacological footprint investigated in this study. Consequently, the lack of comprehensive sample characterization restricts the translational potential of this research. The lack of randomization and data FAIRness in many datasets was problematic. The minimum standards for omics data often result in poor usability due to incomplete characterization of the experimental design and execution, as well as the lack of description regarding potential systematic effects caused by reagents, microarrays, and other factors [58].

### Case Study 2: Multi-omics network integration uncovers epigenetic and transcriptomic regulation of macrophage polarization

This case study demonstrates the potential of MUUMI to integrate multi-omics datasets. Integrating multi-omics data is essential to gain a comprehensive understanding of complex biological systems, as it reveals the intricate regulatory mechanisms operating across different molecular layers. Combining transcriptomics, epigenomics, proteomics, and other omics layers provides insights into biological system regulation that single-omics approaches may overlook [59].

### Experimental setup and data used

In this case study, we analyzed publicly available bulk RNA-Seq and DNA methylation array data previously collected from NCBI Gene Expression Omnibus (GEO), reported with the GEOID: *GSE273628*. In this study, Migliaccio et al. [60] explored the regulatory relationship between DNA methylation and gene expression in macrophages exposed to different polarization stimuli. Cells were differentiated with 30.9 ng/ml of phorbol 12-myristate 13-acetate (PMA) for 48 h. After PMA differentiation, cells are exposed to two different cytokines cocktails and fresh media for 24 h, 48 h, and 72 h. In particular, to simulate a pro-inflammatory environment the cells are exposed to LPS 10 pg/ml and INFγ 20 ng/ml; to induce an anti-inflammatory phenotype instead, the cells are exposed to IL-13 20 ng/ml and IL-4 20 ng/ml.

To gain a comprehensive understanding of the capabilities of the tool and its potential to uncover mechanisms underlying molecular responses to different polarization stimuli, we analyzed the epigenomic and transcriptomic profiles of macrophages stimulated with LPS-INFγ and IL4-IL13. To comply with the findings of Migliaccio et al. 2024, which emphasize the slower kinetics of DNA methylation compared to transcriptional changes, we selected the 72-hour time point for our analysis. This timeframe allows for the detection of more stable epigenetic modifications, including those that may have originated at earlier time points.

### Multi-omics data integration through similarity network fusion

To integrate these datasets, we utilized the *snf_based_integration* function implemented in MUUMI. Through our analysis, we generated two fusion networks: one for LPS-INFγ and one for IL4-IL13-treated macrophages. Such networks are completely connected, and each edge represents the similarity values between genes. The similarity parameter assesses the degree of concordance between the expression patterns of gene pairs in both the transcriptomic and epigenomic layers. A higher similarity score suggests that the genes in a pair tend to exhibit similar regulatory patterns in both transcriptomics and epigenomics layers. Both of the networks are composed of 12,940 nodes and 163,443,600 edges. To retain only the most biologically meaningful connections and make the computation faster, we pruned both of the networks through the function *prune_snf_network*, by setting the 90th percentile as a threshold of significance. The rationale for setting this pruning threshold is reported in Additional File 2. The pruned LPS-INFγ network encompasses 8,502,486 edges while the IL4-IL13 one 8,500,640 edges.

**Fig. 7:**
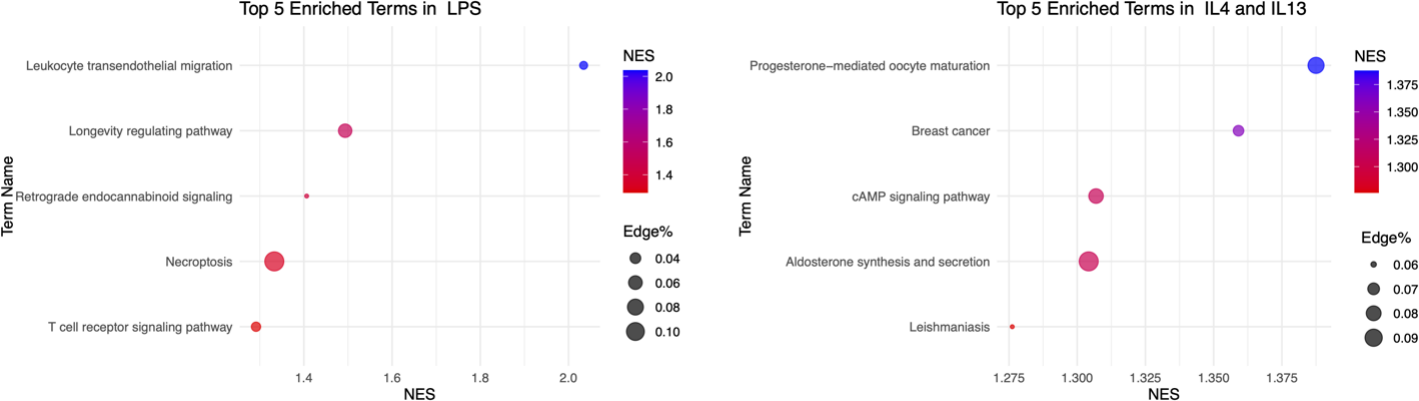
Results of the edge set enrichment analysis (ESEA) on the multi-omics integrated networks of LPS-INFγ-treated macrophages (panel A) and the IL4-IL13-treated macrophages (panel B) (FDR < 0.01)

Subsequently, we ranked the network edges based on their weights and performed a functional annotation through ESEA (Enrichment Set Enrichment Analysis) using KEGG pathways. By performing an enrichment analysis on edges with high similarity scores, we can uncover biological processes that are strongly coordinated at gene expression and epigenetic level. To identify the most significant biological processes and pathways associated with each condition, we performed separate enrichment analysis on the LPS-INFγ and IL4-IL13 networks. The background used in this analysis is composed of 2,087,012 edges divided into 343 edge sets, each representing a biological pathway. To highlight the biological differences between the two exposures, we report only exposure-specific enriched pathways (FDR < 0.01) in Fig. 7.

Our integrated network analysis of the LPS–IFNγ condition reveals a strong correlation of genes involved in T-cell receptor signaling pathways and leukocyte transendothelial migration across the two omics. As expected, these pathways are consistent with a pro-inflammatory phenotype where LPS and IFNγ cooperatively amplify immune-cell activation and recruitment [61, 62] and it aligns well with previous observations reported in Migliaccio et al.

Conversely, the enrichment of the cAMP signaling pathway in the IL4–IL13 condition is consistent with an anti-inflammatory phenotype, as elevated cAMP suppresses NF-κB activity [63] and limits T-cell proliferation [64], supporting that the observed transcriptional and epigenetic patterns reflect a shift toward an inflammation-resolving state. Additionally, the enrichment of aldosterone synthesis and secretion pathways, known to promote extracellular matrix remodeling and fibrosis [65], further supports the interpretation of this phenotype as one that may favor long-term tissue structural repair.

Taken together, these results show that the observed pathway enrichments are driven by tightly co-correlated gene pairs across both transcriptomic and epigenomic layers. This high inter-omic correlation provides a coherent and biologically consistent picture of the inflammatory and anti-inflammatory phenotypes, highlighting pathways that are reinforced at both epigenetic and transcriptional level. By facilitating network-based meta-analyses, MUUMI offers a versatile platform that enables researchers to identify crucial biological pathways, uncover synergistic regulatory effects, and effectively pinpoint key molecular targets. By employing the network analysis features of MUUMI, researchers can integrate omics networks, providing valuable insights into gene sets exhibiting high concordance in all regulatory layers.

### Comparison of MUUMI with existing tools

In this study, we compared the functionalities of MUUMI with other similar packages or web services dedicated to meta-analysis and integration of omics data. We report here a comparison with 7 tools that belong to the same thematic area of MUUMI. Note that the list of tools is not comprehensive.

While a plethora of omics data integration tools exist, a unified framework that encompasses statistical meta-analysis and network integration for diverse omics data does not exist, yet. Existing tools often lack the flexibility for users to customize analytical pipelines to suit specific research questions. This limitation stems from restricted code accessibility, rigid pipelines, and inflexible file formats. Among the investigated tools, MetaOmics, developed by [66], is the closest to MUUMI in terms of functionality but is limited to transcriptomics. It includes modules like MetaQC (quality control), MetaDE (differential expression), MetaPath (pathway enrichment), MetaNetwork (co-expression networks), MetaPredict (predictive modeling), MetaClust (clustering), and MetaPCA (dimensionality reduction). Despite its comprehensive features, it lacks tools for topological network analysis and centrality-based gene ranking. We report a more in-depth benchmark between MUUMI and the MetaDE module of MetaOmics in Additional file 2.

MixOmics [67] is an R package for multi-omics integration using methods like Principal Component Analysis (PCA), Partial Least Squares (PLS), Canonical Correlation Analysis (CCA), and DIABLO (Data Integration Analysis for Biomarker discovery using Latent variable approaches for Omics studies). It supports feature selection, classification, clustering, and visualization, but doesn’t exploit network topology for biological interpretation.

Mergeomics [68] enables integration of multi-omics data using pre-existing networks. It features Marker Dependency Filtering (MDF), Marker Set Enrichment Analysis (MSEA), Key Driver Analysis (KDA), and drug prediction, useful in systems pharmacology. However, it lacks built-in network inference. We carried out a direct comparison of MUUMI with the Mergeomics KDA module to benchmark their driver extrapolation capabilities. The results are showed in Additional file 2.

MergeOmics focuses on integrating multidimensional data using a pre-existing network. Its key features include MDF (dependency filtering), MSEA (pathway enrichment), KDA (key driver analysis), and drug prediction capabilities, especially relevant for drug discovery and repurposing.

IntegrOmics [69] provides tools for integrative analysis of transcriptomics, proteomics, and metabolomics, with a strong emphasis on user-friendly interfaces and visualization. It facilitates tasks like principal component analysis, correlation analysis, and pathway exploration. However, it focuses more on exploratory data analysis and lacks deep support for network-based meta-analysis or high-level pipeline customization.

SNFtool (1) integrates multiple types of omics data by constructing sample similarity networks for each data type and fusing them into a single network. This approach helps identify subtypes or clusters in complex datasets. While powerful for unsupervised learning, SNFtool is limited to sample-level analysis and does not support functional interpretation or gene-level network analysis.

The Netmeta package [70] is a R tool designed for network meta-analysis, which extends traditional meta-analysis by comparing multiple treatments simultaneously across a network of studies. Supports frequentist network meta-analysis for comparing treatments, integrating direct and indirect evidence. Though powerful in clinical meta-analysis, it is not designed for omics data and does not support biological network interpretation.

MOFA (Multi-Omics Factor Analysis) [71] is a statistical framework designed for the integration and dimensionality reduction of multi-omics data in an unsupervised fashion. The tool applies Bayesian factor analysis for unsupervised integration and dimensionality reduction of multi-omics data. MOFA has been recently updated with a new version, named MOFA+ [72]. Compared with MUUMI, MOFA and MOFA + do not include functions for the inference, analysis and interpretation of molecular networks, neglecting the importance of the relationships among the molecular entities under investigation.

Compared with the aforementioned tools, MUUMI balances ease of usage, flexibility of the pipelines and integration of several algorithms that offer a plethora of functionalities to the research community. In fact, MUUMI integrates both statistical meta-analysis with network-based methods for integration of multi-omics data. On one hand, MUUMI combines well-established statistical meta-analysis techniques (widely used beyond the omics domain) with innovative strategies for selecting biologically relevant features. On the other hand, it implements robust tools for the inference and analysis of biological networks to facilitate the interpretation of high-dimensional datasets at both single- and multi-omics level. Notably, MUUMI is the only tool among those compared that implements an Edge Set Enrichment Analysis (ESEA), allowing for the evaluation of the biological relevance of molecular interactions and offering high-resolution insights into the topology of network models. This feature, alongside MUUMI’s capacity to unify different analyses scattered across existing packages and web services, underlines its novelty and practical value. The four-module architecture of our package supports a streamlined workflow from statistical meta-analysis, through network inference and topology-aware annotation, to integration across multiple studies and omics types. Moreover, MUUMI integrates functionalities for batch effect mitigation, network module aggregation and annotation, providing a flexible, interpretable, and systems-level meta-analysis framework for omics data. While many available R packages target specific elements of the multi-omics integration process, MUUMI uniquely merges these capabilities into a single, cohesive environment, addressing critical gaps in current multi-omics meta-analysis platforms. Table 4 summarizes the comparison of tools, highlighting the presence or absence of key features for each tool.

**Table 4:**
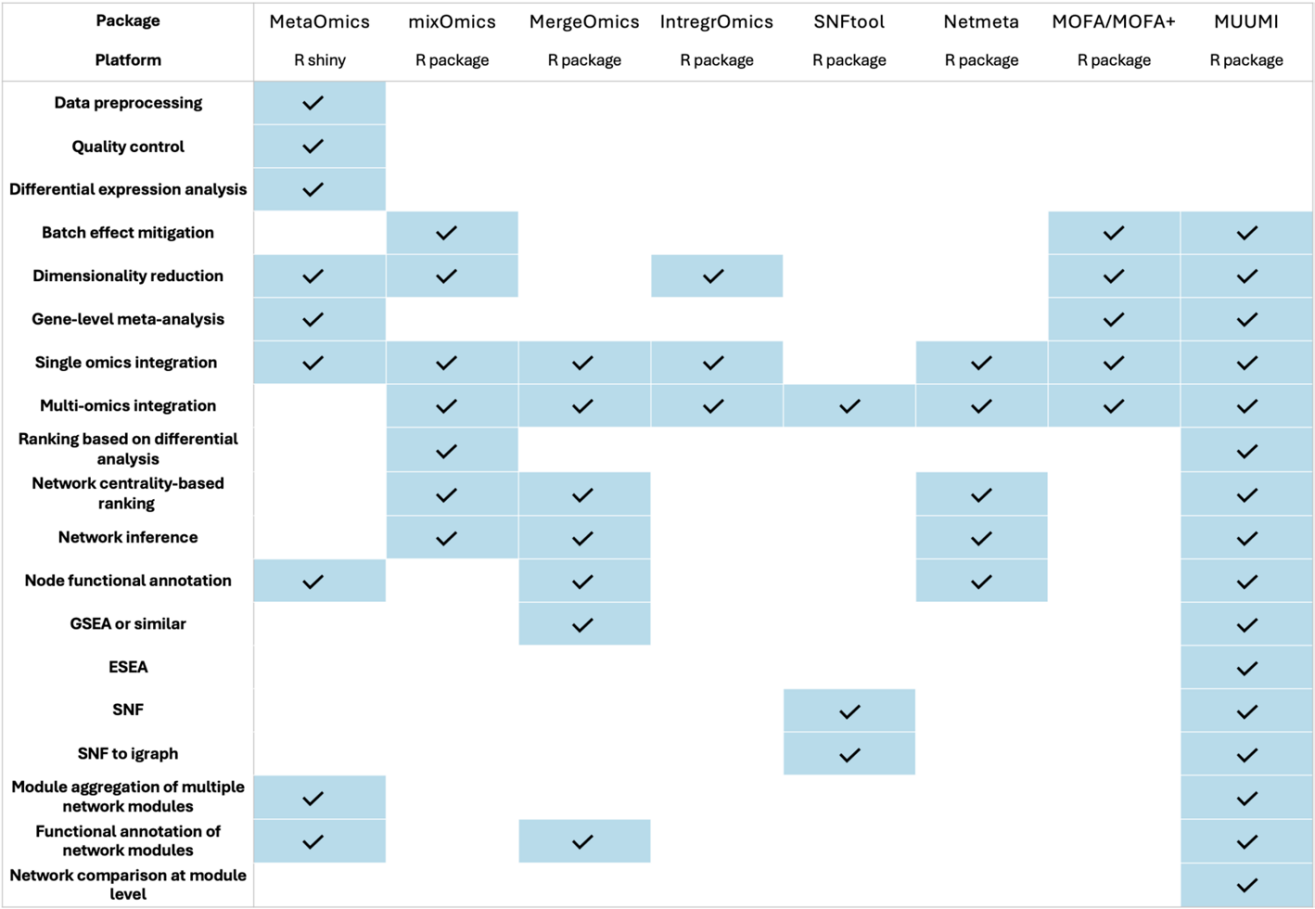
Comparison of MUUMI with other similar tools

## Discussion

Despite many methodologies for multi-omics data integration and meta-analysis have been developed, translating these efforts into biologically meaningful, mechanistic insights remains challenging. Existing softwares focus on isolated aspects of meta-analysis, disregarding the power of network-based approaches to give an holistic view of the biological system or condition under investigation. Other tools provide complex network-based methodologies, but lack functions to aid a robust interpretation of the results. These limitations collectively hinder the exploitation of omics data integration to generate actionable biological insights.

We developed MUUMI to address this gap, by integrating multiple meta-analytical approaches with a comprehensive suite of network-based functionalities, spanning from the inference to the analysis and interpretation of the networks. Moreover, the capacity of MUUMI to handle both single-omics and multi-omics datasets facilitates a more complete interpretation of complex biological systems, providing deeper insights into disease mechanisms, gene regulation, or biomarker discovery. MUUMI includes several exposed functions that can be combined in different ways so as to allow a full customisation of the analysis pipelines according to the biological condition under investigation. In addition, the network-driven interpretation steps, including module functional annotation and the topological enrichment analyses, provide valuable tools to evaluate and explore the biological relevance of the results.

The two case studies presented, an integrative analysis of IPF datasets across RNA-Seq and microarray platforms, and a multi-omics study of macrophage plasticity upon polarising stimuli, demonstrate how MUUMI can reveal biologically plausible insights while accommodating heterogeneous data sources.

## Conclusions

We developed MUUMI, an R package leveraging omics data meta-analysis, integration and interpretation that implements traditional and network-based approaches to unleash the power of multi-study datasets. Statistical and network-based approaches are integrated in a unique framework, allowing the user to derive robust and biologically meaningful results from different studies. The applicability and the functionalities of MUUMI were showcased in the performed case studies.

## Supplementary Information

Below is the link to the electronic supplementary material.

Supplementary Material 1

Supplementary Material 2

## Data Availability

MUUMI is an open-source package and is freely available at [https://github.com/fhaive/muumi](https:/github.com/fhaive/muumi) . For case study 1, the list of publicly available datatsets analysed in this study and direct links to their repositories are reported in Table 1. The pre-processed data and code to reproduce the case studies are available on Zenodo ( [https://zenodo.org/records/15019060](https://zenodo.org/records/15019060) ). For case study 2, the repository and specific dataset utilised are reported within the text. The code to reproduce the case studies is also available on GitHub ( [https://github.com/fhaive/muumi/blob/master/case/_study.R](https://github.com/fhaive/muumi/blob/master/case/_study.R) ).

## Abbreviations

AD: Aggregate data
CCA: Canonical correlation analysis
COL: Collagen genes (e.g., COL1A2, COL3A1, COL14A1, COL15A1)
DIABLO: Data integration analysis for biomarker discovery using latent variable approaches for omics studies
ECM: Extracellular matrix
ESEA: Edge set enrichment analysis
GSEA: Gene set enrichment analysis
IFNγ: Interferon gamma
IL: Interleukin (e.g., IL4, IL13)
IPD: Individual participant data
IPF: Idiopathic pulmonary fibrosis
KDA: Key driver analysis
LPS: Lipopolysaccharide
MDF: Marker dependency filtering
MMP: Matrix metalloproteinase (e.g., MMP7)
MOFA / MOFA+: Multi–omics factor analysis (+ updated version)
MSEA: Marker set enrichment analysis
NMD: Nonsense–mediated mRNA decay
NMF: Non–negative matrix factorization
NF: κB–Nuclear Factor kappa–light–chain–enhancer of activated B cells
ORA: Overrepresentation analysis
PCA: Principal component analysis
PF: Pulmonary fibrosis
PLS: Partial least squares
RNA: Seq–RNA sequencing
SNF: Similarity network fusion
TLR: Toll–like receptor
TNF: Tumor necrosis factor
TNF: α–tumor necrosis factor alpha

## Availability and requirements

Project name: MUUMI. Project home page: https://github.com/fhaive/muumi. Operating system(s): Platform independent. Programming language: R. Other requirements: R version 4.4.1 or higher. License: GPL-3. Any restrictions to use by non-academics: For commercial use and modifications please contact the corresponding author.

## References

[1] Wang B, Mezlini AM, Demir F, Fiume M, Tu Z, Brudno M, et al. Similarity network fusion for aggregating data types on a genomic scale. Nat Methods. 2014;11(3):333–7. ‘. 24464287 10.1038/nmeth.2810

[2] Riley RD, Lambert PC, Abo-Zaid G. Meta-analysis of individual participant data: rationale, conduct, and reporting. BMJ. 2010;340:c221. 20139215 10.1136/bmj.c221

[3] Federico A, Möbus L, Al-Abdulraheem Z, Pavel A, Fortino V, del Giudice G, et al. Integrative network analysis suggests prioritised drugs for atopic dermatitis. J Transl Med. 2024;22(1):64. 38229087 10.1186/s12967-024-04879-4 PMC10792836

[4] Borenstein M, Hedges LV. Effect Sizes for Meta-Analysis. In: Cooper H, Hedges LV, Valentine JC, editors. The handbook of research synthesis and meta-analysis. Russell Sage Foundation; 2019. pp. 207–44.

[5] Kost JT, McDermott MP. Combining dependent *P*-values. Stat Probab Lett. 2002;60(2):183–90.

[6] Breitling R, Armengaud P, Amtmann A, Herzyk P. Rank products: a simple, yet powerful, new method to detect differentially regulated genes in replicated microarray experiments. FEBS Lett. 2004;573(1):83–92. 15327980 10.1016/j.febslet.2004.07.055

[7] Del Carratore F, Jankevics A, Eisinga R, Heskes T, Hong F, Breitling R. RankProd 2.0: a refactored bioconductor package for detecting differentially expressed features in molecular profiling datasets. Bioinformatics. 2017;33(17):2774–5. 28481966 10.1093/bioinformatics/btx292 PMC5860065

[8] Marwah VS, Kinaret PAS, Serra A, Scala G, Lauerma A, Fortino V, et al. Inform: inference of network response modules. Bioinformatics. 2018;34(12):2136–8. 29425308 10.1093/bioinformatics/bty063 PMC9881608

[9] Lee DD, Seung HS. Learning the parts of objects by non-negative matrix factorization. Nature. 1999;401(6755):788–91. 10548103 10.1038/44565

[10] Greene D, Cunningham P. A matrix factorization approach for integrating multiple data views. In: Buntine W, Grobelnik M, Mladenić D, Shawe-Taylor J, editors. Machine Learning and Knowledge Discovery in Databases. Berlin, Heidelberg: Springer; 2009. pp. 423–38.

[11] Di Lieto E, Serra A, Inkala SI, Saarimäki LA, del Giudice G, Fratello M, et al. Esperanto: a GLP-field semi-supervised toxicogenomics metadata curation tool. Bioinformatics. 2023;39(6):btad405. 37354497 10.1093/bioinformatics/btad405 PMC10313344

[12] Furusawa H, Cardwell JH, Okamoto T, Walts AD, Konigsberg IR, Kurche JS et al. Chronic Hypersensitivity Pneumonitis (CHP), an Interstitial Lung Disease (ILD) with Distinct Molecular Signatures [Internet]. Rochester, NY: Social Science Research Network; 2020 [cited 2025 Nov 27]. Available from:

[13] Jaffar J, Wong M, Fishbein GA, Alhamdoosh M, McMillan L, Gamell-Fulla C, et al. Matrix metalloproteinase-7 is increased in lung bases but not apices in idiopathic pulmonary fibrosis. ERJ Open Res. 2022;8(4):00191–2022. 36299365 10.1183/23120541.00191-2022 PMC9589331

[14] McDonough JE, Ahangari F, Li Q, Jain S, Verleden SE, Herazo-Maya J, et al. Transcriptional regulatory model of fibrosis progression in the human lung. JCI Insight. 2019;4(22):e131597. 31600171 10.1172/jci.insight.131597 PMC6948862

[15] Huang Y, Guzy R, Ma SF, Bonham CA, Jou J, Schulte JJ, et al. Central lung gene expression associates with myofibroblast features in idiopathic pulmonary fibrosis. BMJ Open Respir Res. 2023;10(1):e001391. 36725082 10.1136/bmjresp-2022-001391 PMC9896241

[16] Schafer M, White T, Iijima K, Mazula D, Passos J, Kirkland JL, et al. Cellular senescence drives fibrotic pulmonary disease. Innov Aging. 2017;1(Suppl 1):135.

[17] DePianto DJ, Chandriani S, Abbas AR, Jia G, N’Diaye EN, Caplazi P, et al. Heterogeneous gene expression signatures correspond to distinct lung pathologies and biomarkers of disease severity in idiopathic pulmonary fibrosis. Thorax. 2015;70(1):48–56. 25217476 10.1136/thoraxjnl-2013-204596 PMC4472447

[18] DePianto DJ, Heiden JAV, Morshead KB, Sun KH, Modrusan Z, Teng G, et al. Molecular mapping of interstitial lung disease reveals a phenotypically distinct senescent basal epithelial cell population. JCI Insight. 2021;6(8):e143626. 33705361 10.1172/jci.insight.143626 PMC8119199

[19] Cecchini MJ, Hosein K, Howlett CJ, Joseph M, Mura M. Comprehensive gene expression profiling identifies distinct and overlapping transcriptional profiles in non-specific interstitial pneumonia and idiopathic pulmonary fibrosis. Respir Res. 2018;19(1):153. 30111332 10.1186/s12931-018-0857-1 PMC6094889

[20] Cho JH, Gelinas R, Wang K, Etheridge A, Piper MG, Batte K, et al. Systems biology of interstitial lung diseases: integration of mRNA and microRNA expression changes. BMC Med Genomics. 2011;4:8. 21241464 10.1186/1755-8794-4-8 PMC3035594

[21] Meltzer EB, Barry WT, D’Amico TA, Davis RD, Lin SS, Onaitis MW, et al. Bayesian probit regression model for the diagnosis of pulmonary fibrosis: proof-of-principle. BMC Med Genomics. 2011;4:70. 21974901 10.1186/1755-8794-4-70 PMC3199230

[22] Geng J, Huang X, Li Y, Xu X, Li S, Jiang D, et al. Down-regulation of USP13 mediates phenotype transformation of fibroblasts in idiopathic pulmonary fibrosis. Respir Res. 2015;16:124. 26453058 10.1186/s12931-015-0286-3 PMC4600336

[23] Brereton CJ, Yao L, Davies ER, Zhou Y, Vukmirovic M, Bell JA, et al. Pseudohypoxic HIF pathway activation dysregulates collagen structure-function in human lung fibrosis. Elife. 2022;11:e69348. 35188460 10.7554/eLife.69348 PMC8860444

[24] Luzina IG, Salcedo MV, Rojas-Peña ML, Wyman AE, Galvin JR, Sachdeva A, et al. Transcriptomic evidence of immune activation in macroscopically normal-appearing and scarred lung tissues in idiopathic pulmonary fibrosis. Cell Immunol. 2018;325:1–13. 29329637 10.1016/j.cellimm.2018.01.002 PMC5826809

[25] Konishi K, Gibson KF, Lindell KO, Richards TJ, Zhang Y, Dhir R, et al. Gene expression profiles of acute exacerbations of idiopathic pulmonary fibrosis. Am J Respir Crit Care Med. 2009;180(2):167–75. 19363140 10.1164/rccm.200810-1596OC PMC2714820

[26] Yin Q, Strong MJ, Zhuang Y, Flemington EK, Kaminski N, de Andrade JA, et al. Assessment of viral RNA in idiopathic pulmonary fibrosis using RNA-seq. BMC Pulm Med. 2020;20(1):81. 32245461 10.1186/s12890-020-1114-1 PMC7119082

[27] King TE, Pardo A, Selman M. Idiopathic pulmonary fibrosis. Lancet. 2011;378(9807):1949–61. 21719092 10.1016/S0140-6736(11)60052-4

[28] Martinez FJ, Collard HR, Pardo A, Raghu G, Richeldi L, Selman M, et al. Idiopathic pulmonary fibrosis. Nat Rev Dis Primers. 2017;3:17074. 29052582 10.1038/nrdp.2017.74

[29] Wuyts WA, Agostini C, Antoniou KM, Bouros D, Chambers RC, Cottin V, et al. The pathogenesis of pulmonary fibrosis: a moving target. Eur Respir J. 2013;41(5):1207–18. 23100500 10.1183/09031936.00073012

[30] Misharin AV, Morales-Nebreda L, Reyfman PA, Cuda CM, Walter JM, McQuattie-Pimentel AC, et al. Monocyte-derived alveolar macrophages drive lung fibrosis and persist in the lung over the life span. J Exp Med. 2017;214(8):2387–404. 28694385 10.1084/jem.20162152 PMC5551573

[31] Saarimäki LA, Kinaret PAS, Scala G, del Giudice G, Federico A, Serra A, et al. Toxicogenomics analysis of dynamic dose-response in macrophages highlights molecular alterations relevant for multi-walled carbon nanotube-induced lung fibrosis. NanoImpact. 2020;20:100274.

[32] Sugeir S, De Moraes AG. Bronchoscopy in the Intensive Care Unit. In: Demetriades D, Inaba K, Lumb PD, editors. Atlas of Critical Care Procedures [Internet]. Cham: Springer International Publishing; 2018 [cited 2025 Nov 27]. pp. 49–55. Available from:

[33] Love MI, Huber W, Anders S. Moderated estimation of fold change and dispersion for RNA-seq data with DESeq2. Genome Biol. 2014;15(12):550. 25516281 10.1186/s13059-014-0550-8 PMC4302049

[34] Marwah VS, Scala G, Kinaret PAS, Serra A, Alenius H, Fortino V, et al. eUTOPIA: solution for omics data preprocessing and analysis. Source Code Biol Med. 2019;14(1):1. 30728855 10.1186/s13029-019-0071-7 PMC6352382

[35] Ritchie ME, Phipson B, Wu D, Hu Y, Law CW, Shi W, et al. limma powers differential expression analyses for RNA-sequencing and microarray studies. Nucleic Acids Res. 2015;43(7):e47. 25605792 10.1093/nar/gkv007 PMC4402510

[36] Benjamini Y, Hochberg Y. Controlling the false discovery rate: a practical and powerful approach to multiple testing. Royal Stat Soc J Ser B: Methodological. 1995;57(1):289–300.

[37] Zolak JS, de Andrade JA. Idiopathic pulmonary fibrosis. Immunol Allergy Clin North Am. 2012;32(4):473–85. 23102062 10.1016/j.iac.2012.08.006

[38] Zhou Z, Qu J, He L, Zhu Y, Yang SZ, Zhang F, et al. Stiff matrix instigates type I collagen biogenesis by mammalian cleavage factor I complex-mediated alternative polyadenylation. JCI Insight. 2020;5(3):e133972. 31935199 10.1172/jci.insight.133972 PMC7098798

[39] Mei Q, Liu Z, Zuo H, Yang Z, Qu J. Idiopathic pulmonary fibrosis: an update on pathogenesis. Front Pharmacol. 2021;12:797292. 35126134 10.3389/fphar.2021.797292 PMC8807692

[40] Huang S, Lai X, Yang L, Ye F, Huang C, Qiu Y, et al. Asporin promotes TGF-β-induced lung myofibroblast differentiation by facilitating Rab11-dependent recycling of TβRI. Am J Respir Cell Mol Biol. 2022;66(2):158–70. 34705621 10.1165/rcmb.2021-0257OC

[41] Frangogiannis N. Transforming growth factor-β in tissue fibrosis. J Exp Med. 2020;217(3):e20190103. 32997468 10.1084/jem.20190103 PMC7062524

[42] Bauer Y, White ES, de Bernard S, Cornelisse P, Leconte I, Morganti A, et al. MMP-7 is a predictive biomarker of disease progression in patients with idiopathic pulmonary fibrosis. ERJ Open Res. 2017;3(1):00074–2016. 28435843 10.1183/23120541.00074-2016 PMC5395293

[43] Mahalanobish S, Saha S, Dutta S, Sil PC. Matrix metalloproteinase: An upcoming therapeutic approach for idiopathic pulmonary fibrosis. Pharmacol Res. 2020;152:104591. 31837390 10.1016/j.phrs.2019.104591

[44] Yu G, Tzouvelekis A, Wang R, Herazo-Maya JD, Ibarra GH, Srivastava A, et al. Thyroid hormone inhibits lung fibrosis in mice by improving epithelial mitochondrial function. Nat Med. 2018;24(1):39–49. 29200204 10.1038/nm.4447 PMC5760280

[45] Liberzon A, Subramanian A, Pinchback R, Thorvaldsdóttir H, Tamayo P, Mesirov JP. Molecular signatures database (MSigDB) 3.0. Bioinformatics. 2011;27(12):1739–40. 21546393 10.1093/bioinformatics/btr260 PMC3106198

[46] Åhrman E, Hallgren O, Malmström L, Hedström U, Malmström A, Bjermer L, et al. Quantitative proteomic characterization of the lung extracellular matrix in chronic obstructive pulmonary disease and idiopathic pulmonary fibrosis. J Proteom. 2018;189:23–33. 10.1016/j.jprot.2018.02.027 29501846

[47] Qian W, Xia S, Yang X, Yu J, Guo B, Lin Z, et al. Complex involvement of the extracellular matrix, immune effect, and lipid metabolism in the development of idiopathic pulmonary fibrosis. Front Mol Biosci. 2021;8:800747. 35174208 10.3389/fmolb.2021.800747 PMC8841329

[48] Federico A, Hautanen V, Christian N, Kremer A, Serra A, Greco D. Manually curated and harmonised transcriptomics datasets of psoriasis and atopic dermatitis patients. Sci Data. 2020;7(1):343. 33051456 10.1038/s41597-020-00696-8 PMC7555498

[49] Hastie T, Tibshirani R, Balasubramanian N, Gil C. pamr: Pam: Prediction Analysis for Microarrays [Internet]. Comprehensive R Archive Network (CRAN); 2019 [cited 2025 Nov 27]. Available from: https://CRAN.R-project.org/package=pamr

[50] Pons P, Latapy M. Computing communities in large networks using random walks (long version) [Internet]. arXiv; 2005. Available from: http://arxiv.org/abs/physics/0512106

[51] Reichardt J, Bornholdt S. Statistical mechanics of community detection. Phys Rev E. 2006;74(1):016110. 10.1103/PhysRevE.74.016110 16907154

[52] Newman MEJ, Girvan M. Finding and evaluating community structure in networks. Phys Rev E. 2004;69(2):026113. 10.1103/PhysRevE.69.026113 14995526

[53] Blondel VD, Guillaume JL, Lambiotte R, Lefebvre E. Fast unfolding of communities in large networks. J Stat Mech. 2008;2008(10):P10008.

[54] Clauset A, Newman MEJ, Moore C. Finding community structure in very large networks. Phys Rev E. 2004;70(6):066111. 10.1103/PhysRevE.70.066111 15697438

[55] Butler MW, Keane MP. The Role of Immunity and Inflammation in IPF Pathogenesis. In: Meyer KC, Nathan SD, editors. Idiopathic pulmonary fibrosis: a comprehensive clinical guide. Springer International Publishing; 2019. pp. 97–131.

[56] Upagupta C, Shimbori C, Alsilmi R, Kolb M. Matrix abnormalities in pulmonary fibrosis. Eur Respir Rev. 2018;27(148):180033. 29950306 10.1183/16000617.0033-2018 PMC9489108

[57] Hara H, Kuwano K, Araya J. Mitochondrial Quality Control in COPD and IPF. Cells. 2018;7(8):86. 30042371 10.3390/cells7080086 PMC6115906

[58] Saarimäki LA, Melagraki G, Afantitis A, Lynch I, Greco D. Prospects and challenges for FAIR toxicogenomics data. Nat Nanotechnol. 2022;17(1):17–8. 34949777 10.1038/s41565-021-01049-1

[59] Hasin Y, Seldin M, Lusis A. Multi-omics approaches to disease. Genome Biol. 2017;18(1):83. 28476144 10.1186/s13059-017-1215-1 PMC5418815

[60] Migliaccio G, Morikka J, Del Giudice G, Vaani M, Möbus L, Serra A, et al. Methylation and transcriptomic profiling reveals short term and long term regulatory responses in polarized macrophages. Comput Struct Biotechnol J. 2024;25:143–52. 39257962 10.1016/j.csbj.2024.08.018 PMC11385784

[61] Murray PJ, Allen JE, Biswas SK, Fisher EA, Gilroy DW, Goerdt S, et al. Macrophage activation and polarization: nomenclature and experimental guidelines. Immunity. 2014;41(1):14–20. 25035950 10.1016/j.immuni.2014.06.008 PMC4123412

[62] Lawrence T, Natoli G. Transcriptional regulation of macrophage polarization: enabling diversity with identity. Nat Rev Immunol. 2011;11(11):750–61. 22025054 10.1038/nri3088

[63] Chen S, Saeed AFUH, Liu Q, Jiang Q, Xu H, Xiao GG, et al. Macrophages in immunoregulation and therapeutics. Sig Transduct Target Ther. 2023;8(1):207. 10.1038/s41392-023-01452-1 PMC10200802 37211559

[64] Mosenden R, Taskén K. Cyclic AMP-mediated immune regulation—overview of mechanisms of action in T cells. Cell Signal. 2011;23(6):1009–16. 21130867 10.1016/j.cellsig.2010.11.018

[65] Gilbert KC, Brown NJ. Aldosterone and Inflammation. Curr Opin Endocrinol Diabetes Obes. 2010;17(3):199–204. 20422780 10.1097/med.0b013e3283391989 PMC4079531

[66] Ma T, Huo Z, Kuo A, Zhu L, Fang Z, Zeng X, et al. Metaomics: analysis pipeline and browser-based software suite for transcriptomic meta-analysis. Bioinformatics. 2019;35(9):1597–9. 30304367 10.1093/bioinformatics/bty825 PMC6499246

[67] Rohart F, Gautier B, Singh A, Cao KAL. mixOmics: an R package for ‘omics feature selection and multiple data integration. PLoS Comput Biol. 2017;13(11):e1005752. 29099853 10.1371/journal.pcbi.1005752 PMC5687754

[68] Ding J, Blencowe M, Nghiem T, Ha SM, Chen YW, Li G, et al. Mergeomics 2.0: a web server for multi-omics data integration to elucidate disease networks and predict therapeutics. Nucleic Acids Res. 2021;49(W1):W375–87. 34048577 10.1093/nar/gkab405 PMC8262738

[69] Lê Cao KA, González I, Déjean S. integrOmics: an R package to unravel relationships between two omics datasets. Bioinformatics. 2009;25(21):2855–6. 19706745 10.1093/bioinformatics/btp515 PMC2781751

[70] Balduzzi S, Rücker G, Nikolakopoulou A, Papakonstantinou T, Salanti G, Efthimiou O, et al. netmeta: An R package for network meta-analysis using frequentist methods. J Stat Softw. 2023;106:1–40.37138589

[71] Argelaguet R, Velten B, Arnol D, Dietrich S, Zenz T, Marioni JC, et al. Multi-omics factor analysis—a framework for unsupervised integration of multi‐omics data sets. Mol Syst Biol. 2018;14(6):e8124. 29925568 10.15252/msb.20178124 PMC6010767

[72] Argelaguet R, Arnol D, Bredikhin D, Deloro Y, Velten B, Marioni JC, et al. MOFA+: a statistical framework for comprehensive integration of multi-modal single-cell data. Genome Biol. 2020;21(1):111. 32393329 10.1186/s13059-020-02015-1 PMC7212577

