# Supplementary material for: MUUMI: an R package for statistical and network-based meta-analysis for multi-omics data integration
Source: BMC Bioinformatics. 2026 Feb 3;27:56. doi: 10.1186/s12859-026-06394-3 (PMC12955011; doi:10.1186/s12859-026-06394-3)
Supplement: Supplementary file 2 — Supplementary Material 2 [file 12859_2026_6394_MOESM2_ESM.docx]

**SENSITIVITY ANALYSIS FOR SNF NETWORK PRUNING THRESHOLD DEFINITION**

To determine an appropriate pruning threshold for the fused SNF networks, we performed a systematic sensitivity analysis across a range of candidate cutoffs between the 80^th^–100^th^ percentiles of the edge-weight distribution. Given that SNF produces extremely dense networks, retaining all edges would obscure meaningful biological structure and hinder downstream analyses, such as Edge Set Enrichment Analysis (ESEA). For each candidate threshold, we evaluated 1) changes in global topological properties, including average degree, modularity, and the number of connected components, and 2) the number of significantly enriched pathways detected by ESEA on the pruned networks. Thresholds above the 95^th^ percentile caused network fragmentation and marked increases in modularity, indicating excessive edge removal and loss of coherent biological structure (Figure S1). We therefore focused on the stable range between the 80^th^ and 95^th^ percentiles. Within this interval, the 90th percentile consistently produced a number of enriched pathways closest to the mean enrichment observed across the range, while preserving network connectivity and reducing noise from weak or spurious edges. Based on this combined topological and functional assessment, we selected the 90^th^ percentile as a robust and biologically informed cutoff for pruning the SNF-derived networks (Figure S2).

**
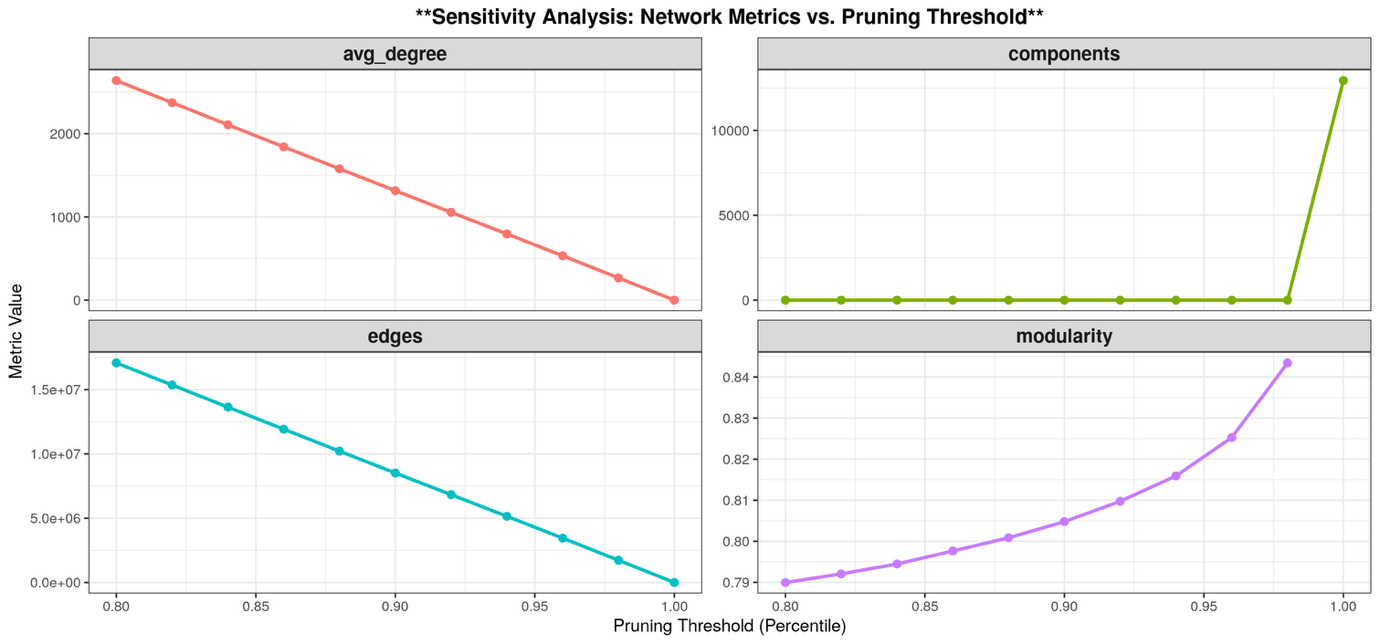
**

*Figure S1 - Sensitivity analysis examining the relationship between topological network metrics and the pruning threshold (percentile). Four key network characteristics are displayed across separate subplots: 1) Average Degree (top-left): shows how the average degree of nodes in the network changes as the pruning threshold increases. Components (top-right): illustrates the number of connected components within the network at different pruning thresholds. Number of edges (bottom-left): depicts the total number of edges remaining in the network as the pruning threshold is varied. Modularity (bottom-right): indicates how the modularity of the network structure evolves with increasing pruning thresholds. In all panels, the x-axis represents the "Pruning Threshold (Percentile)," ranging from 0.80 to 1.00, while the y-axis represents the "Metric Value" for each respective network characteristic. The trends showed in the present figure represent patterns observed in both IL4-IL13 and LPS networks.*

**
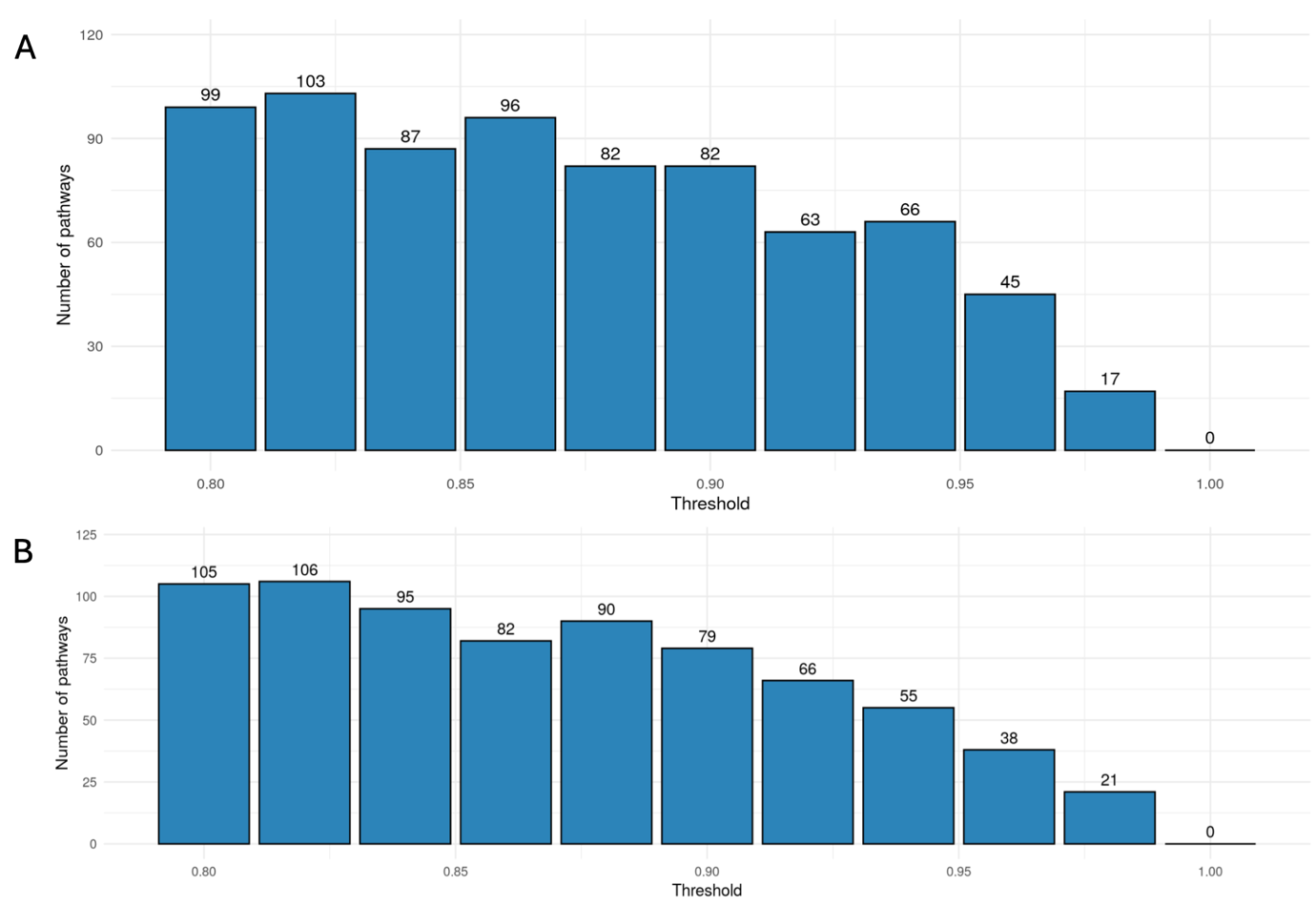
**

*Figure S2 – Number of significantly enriched pathways at each of the selected pruning thresholds in IL4-IL13 (Panel A) and LPS (Panel B) networks.*

**COMPARISON OF MUUMI WITH METAOMICS IN META-ANALYSIS PERFORMANCE**

To compare the meta-analysis results and their consistency between MUUMI and the MetaDE module of MetaOmics, we performed differential-expression meta-analysis. For each dataset, we generated gene-level adjusted p-values and retained only genes shared across all studies.

MetaOmics combines per-study p-values using Fisher’s method, which aggregates the statistical significance across cohorts by summing log-transformed p-values and evaluating the resulting statistic against a chi-square distribution (63). This produces a unified meta-analytic p-value and an associated gene ranking, with lower p-values indicating higher significance. Although MetaOmics also supports Stouffer and adaptively weighted (AW) models, Fisher’s method was used for primary comparison in this study.

MUUMI’s *run_ensembl_metanalysis* function was applied to the same input. In contrast to MetaOmics, MUUMI integrates multiple evidence streams—effect-size meta-statistics, p-value combination (sumlog), and rank-product scores—into a single consensus gene ranking. Whereas MetaOmics reports each model output separately, MUUMI produces a unified prioritization that incorporates complementary information beyond p-values alone.

To compare the two approaches, MUUMI consensus ranks were evaluated against MetaOmics Fisher-based ranks. After harmonizing gene identifiers, 9,371 genes were shared between both pipelines.

MOFA (Multi-Omics Factor Analysis) appears in Table 4 because it supports multi-omics integration; however, MOFA produces latent factors that summarize sample-level variation rather than per-gene meta-analytic statistics (69). It does not combine p-values or provide ranked gene lists and therefore cannot be directly compared with MUUMI or MetaOmics in this setting.

Although several tools in Table 4 share overlapping functional checkmarks with MUUMI, these reflect general analytical capabilities (e.g., multi-omics integration, pathway analysis, clustering) rather than production of comparable gene-level meta-analysis output. Most listed tools yield sample-level results, such as latent factors (MOFA) or component loadings (mixOmics), or function primarily as preprocessing or visualization frameworks. Because these tools do not report ranked per-gene statistics across studies, they cannot be used for direct benchmarking of gene-level meta-analytic performance. Among the listed tools, MetaOmics (MetaDE) is the only one producing directly comparable gene-level meta-analysis output; therefore, it served as the appropriate benchmark. The strong concordance observed between MUUMI and MetaOmics demonstrates that MUUMI recovers the same biological signal as an established method while simultaneously integrating additional layers of evidence.
 **BENCHMARKING MUUMI AGAINST MERGEOMICS wKDA**

To evaluate the robustness of MUUMI’s driver identification, we benchmarked MUUMI against the widely used Mergeomics wKDA algorithm. Both tools were run on the identical IPF biopsy co-expression network and module definitions. MUUMI drivers were defined as the top 100 genes per module based on a Borda-rank across four centrality measures (degree, betweenness, closeness, clustering coefficient), whereas wKDA drivers were defined as the top 100 genes per module ranked by KDA p-values.

Across modules, MUUMI and Mergeomics shared 6–37 driver genes, corresponding to Jaccard indices of 0.06–0.23, which is consistent with expected variability between centrality-based and neighborhood-enrichment-based driver definitions. Importantly, Reactome enrichment of the driver genes revealed substantially stronger agreement at the pathway level. Modules 3, 4, and 6 showed 25–33% overlap among the top 10 enriched pathways, with both approaches highlighting similar biological themes (e.g., neutrophil activation, phagocytosis, cell-cycle regulation, and Rho GTPase signaling). Despite differences in individual driver genes, both methods converged on the same overarching biological processes, indicating that MUUMI captures biologically meaningful regulatory structure within modules. Table S1 shows the statistics about the overlap of the top 100 genes in each module between MUUMI and Mergeomics, while Table S2 focuses on the statistics about the top 10 enriched pathways in the two methods. In Figure S3 are showed the results of the functional annotation obtained from both tools across the network modules.

| module | muumi_n | mergeomics_n | overlap_n | overlap_jaccard |
| --- | --- | --- | --- | --- |
| 1 | 100 | 100 | 17 | 0.09289617 |
| 2 | 100 | 100 | 12 | 0.06382979 |
| 3 | 100 | 100 | 22 | 0.12359551 |
| 4 | 100 | 100 | 26 | 0.14942529 |
| 5 | 100 | 100 | 37 | 0.22699387 |
| 6 | 100 | 100 | 35 | 0.21212121 |

*Table S1 – Comparison statistics between MUUMI and Mergeomics in the top 100 genes in each network module.*

| module | muumi_n | mergeomics_n | overlap_n | overlap_jaccard |
| --- | --- | --- | --- | --- |
| 1 | 10 | 10 | 0 | 0.00000000 |
| 2 | 10 | 10 | 1 | 0.05263158 |
| 3 | 10 | 10 | 5 | 0.33333333 |
| 4 | 10 | 10 | 2 | 0.11111111 |
| 5 | 10 | 10 | 0 | 0.00000000 |
| 6 | 10 | 10 | 4 | 0.25000000 |

*Table S2 – Comparison statistics between MUUMI and Mergeomics in the top 10 biological pathways enriched in each network module.*


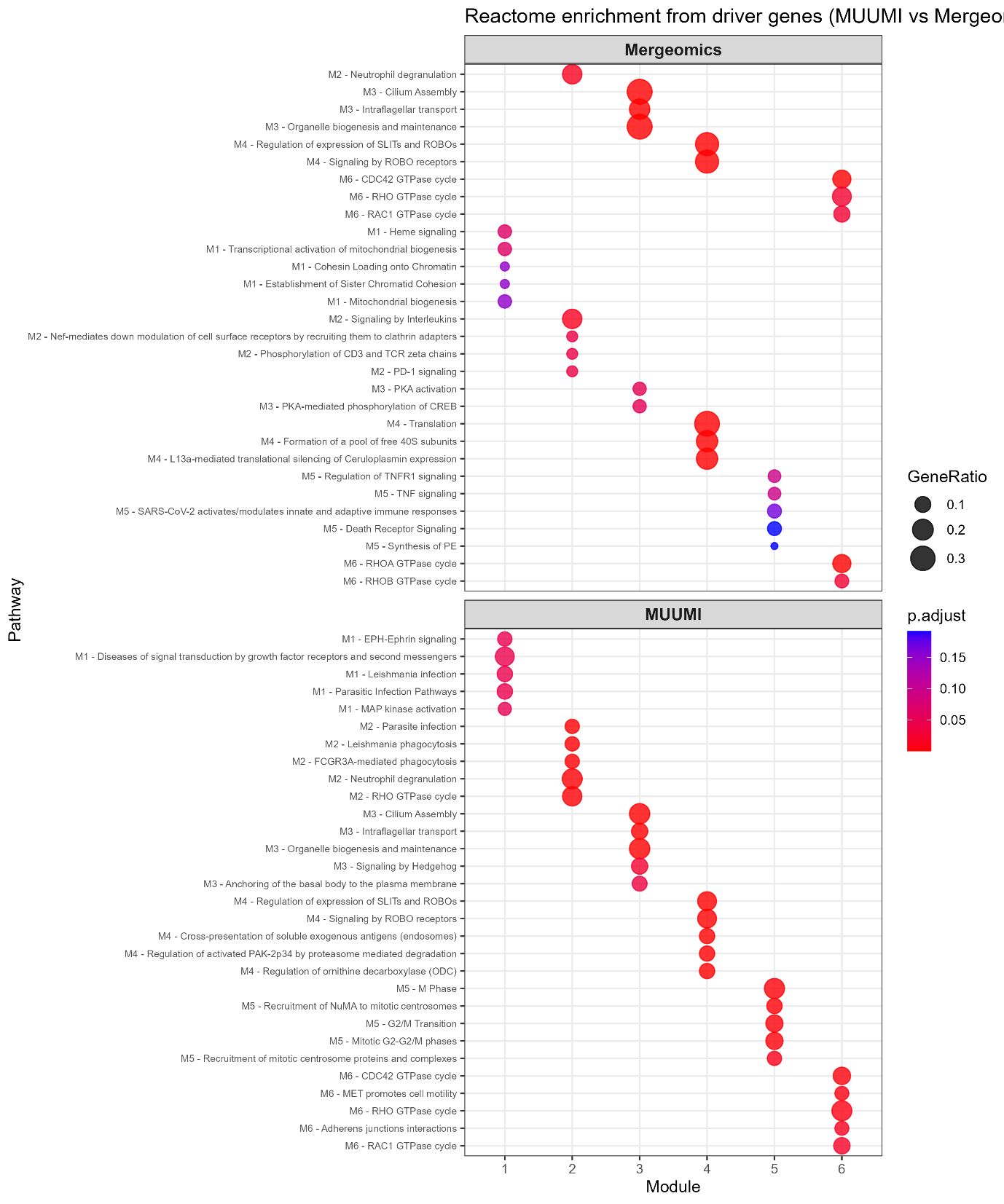


*Figure S3 – Comparison of the functional annotation results between MUUMI and Mergeomics for each of the network modules.*
